# Repurposing Leucovorin for Mild Traumatic Brain Injury: Evidence from Biochemical and Behavioral Outcomes in Rats

**DOI:** 10.3390/ph19060865

**Published:** 2026-05-30

**Authors:** Erdem Arslan, Melike Ordu

**Affiliations:** 1 Department of Medical Pharmacology, Faculty of Medicine, Aksaray University, 68100 Aksaray, Türkiye; 2 Department of Medicinal Pathology, Faculty of Medicine, Aksaray University, 68100 Aksaray, Türkiye; melikeordu@aksaray.edu.tr

**Keywords:** leucovorin, folinic acid, mild traumatic brain injury, oxidative stress, neuroinflammation, recognition memory

## Abstract

**Background/Objectives:** Mild traumatic brain injury (mTBI) is common and may result in persistent cognitive and affective disturbances driven, at least in part, by delayed secondary injury mechanisms, including oxidative stress, neuroinflammation, apoptosis-related signaling, and impaired neuroplasticity. Pharmacological strategies targeting these interconnected processes remain limited. The present study investigated leucovorin, also known as folinic acid, a clinically approved reduced folate, as a potential repurposing candidate in an experimental model of mTBI. **Methods:** Male Wistar rats were subjected to mild diffuse brain injury using a modified weight-drop model and received a single intraperitoneal dose of leucovorin (20 mg/kg). Behavioral performance was evaluated using the open field, elevated plus maze, forced swim, and novel object recognition tests. Oxidative stress markers, including total antioxidant status (TAS), total oxidant status (TOS), and oxidative stress index (OSI), as well as inflammatory mediators tumor necrosis factor-α (TNF-α) and cyclooxygenase-2 (COX-2), caspase-3, brain-derived neurotrophic factor (BDNF), and acetylcholinesterase (AChE), were measured in hippocampal tissue and plasma. Histopathological and immunohistochemical evaluations were also performed in cortical and hippocampal regions. **Results:** Experimental mTBI was associated with anxiety-like and depressive-like behaviors and impaired recognition memory, whereas basal locomotor activity was not significantly altered. Trauma was also associated with increased oxidative stress, elevated inflammatory and apoptosis-related markers, reduced BDNF levels, altered AChE activity, and histopathological abnormalities. Compared with untreated mTBI animals, leucovorin-treated animals showed attenuation of biochemical and tissue alterations, accompanied by improved behavioral outcomes. Immunohistochemical findings were consistent with reduced inflammatory labeling and relative preservation of tissue architecture following leucovorin treatment. **Conclusions:** Leucovorin attenuated behavioral, biochemical, histopathological, and immunohistochemical alterations associated with experimental mTBI. These findings suggest that leucovorin may have neuroprotective potential in this setting; however, further studies are needed to clarify the underlying mechanisms, optimal treatment paradigms, and translational relevance.

## 1. Introduction

Mild traumatic brain injury (mTBI) is the most prevalent form of head trauma and represents a major public health concern, as it may lead to persistent neurocognitive and affective sequelae despite the absence of overt structural injury [1,2,3]. Although classified as “mild,” mTBI is frequently associated with long-term impairments in attention, executive function, information-processing speed, and mood regulation, reflecting sustained disturbances in neuronal network function rather than a purely transient biomechanical event [4,5,6,7].

At the cellular and molecular levels, mTBI initiates a temporally evolving secondary injury cascade that extends well beyond the initial mechanical insult and plays a central role in determining long-term outcomes [8,9,10]. Mechanical deformation of neuronal and glial membranes induces ionic disequilibrium and excessive glutamate release, leading to calcium overload and mitochondrial bioenergetic dysfunction [11,12]. This mitochondrial impairment promotes reactive oxygen species generation and lipid peroxidation, thereby amplifying oxidative stress and rendering neurons vulnerable to downstream apoptosis-related signaling [13,14]. In parallel, damage-associated molecular patterns and cellular stress signals activate microglia and astrocytes, thereby engaging NF-κB-linked transcriptional programs that increase pro-inflammatory mediators, including tumor necrosis factor-α (TNF-α) and cyclooxygenase-2 (COX-2), which further compromise synaptic integrity and neuronal survival [9,15,16]. These oxidative and inflammatory processes can converge on intrinsic apoptosis-related pathways, including caspase-3 activation, thereby contributing to progressive neuronal loss and impaired recovery of learning- and mood-related circuits [10]. Despite extensive characterization of these interrelated mechanisms, no pharmacological therapy has consistently demonstrated efficacy in attenuating secondary injury pathways or improving durable functional outcomes after mTBI [2,6].

One biological system increasingly recognized as relevant to secondary injury biology is folate-dependent one-carbon metabolism, which integrates redox regulation, inflammatory signaling, apoptosis, and epigenetic control of neuroplasticity-related gene expression [17,18,19]. Perturbations in one-carbon flux can exacerbate oxidative stress by limiting endogenous antioxidant capacity and increasing susceptibility to lipid peroxidation, whereas restoration of reduced folate availability has been shown to improve redox balance in experimental systems, commonly reflected by increased total antioxidant status (TAS) and reduced total oxidant status (TOS) and oxidative stress index (OSI) [20,21]. In parallel, folate deficiency is associated with exaggerated neuroinflammatory responses, whereas improved availability of reduced folate has been reported to suppress pro-inflammatory mediators, including TNF-α and COX-2, potentially through modulation of NF-κB–dependent transcriptional pathways [10,22].

Folate metabolism also intersects with apoptotic signaling cascades and neuroplasticity. Experimental studies have shown that folate insufficiency promotes caspase-3–mediated apoptosis and neuronal vulnerability, whereas restoration of reduced folate pools attenuates apoptosis-related signaling and limits neuronal loss [22,23]. Beyond cell survival, one-carbon metabolism contributes to the epigenetic regulation of genes critical for synaptic plasticity, including brain-derived neurotrophic factor (BDNF), thereby influencing learning and memory processes that are frequently disrupted after mTBI [24,25]. In addition, folate-dependent methylation pathways regulate cholinergic homeostasis; folate deficiency has been linked to increased acetylcholinesterase (AChE) activity and impaired cholinergic neurotransmission, whereas restoration of methylation balance may help restore AChE activity and support cognitive function [26,27].

Leucovorin (LEU; folinic acid, calcium folinate) is a clinically established reduced folate that can be rapidly converted into active folate cofactors and thereby support one-carbon metabolic flux without requiring initial dihydrofolate reductase (DHFR)-dependent reduction, making it a practical tool for modulating folate-dependent pathways [28]. Given its long-standing clinical use, including methotrexate rescue and fluoropyrimidine modulation, and its generally favorable safety profile, LEU provides a translationally feasible approach for probing folate-linked mechanisms relevant to secondary injury biology. Reduced folate/one-carbon metabolism has extensive experimental links to redox homeostasis and oxidative injury, inflammatory signaling, apoptosis and DNA damage responses, neurogenesis and plasticity, and cholinergic regulation, often through folate–homocysteine–methionine/S-adenosylmethionine (SAM) and folate–choline coupling [29]. However, despite this broad biological relevance, the specific effects of LEU on these processes in traumatic brain injury (TBI) remain insufficiently defined; to date, only limited preclinical work has directly evaluated folinic acid in experimental head injury, with at least one study focusing primarily on inflammatory and homocysteine-related readouts after treatment [30].

Leucovorin was selected instead of L-methylfolate because the present study was designed as a repurposing investigation of folinic acid, a clinically established reduced folate with available parenteral formulations and long-standing therapeutic use. Unlike folic acid, leucovorin does not require initial dihydrofolate reductase-dependent reduction before entering active folate-dependent one-carbon metabolism [28,31]. Although L-methylfolate is the biologically active methylated folate form and has recognized CNS relevance, its current evidence base is stronger in psychiatric and monoaminergic contexts than in experimental TBI [32,33,34]. In contrast, limited preclinical evidence has directly evaluated folinic acid in a head injury model and reported reductions in homocysteine- and inflammation-related markers [30]. Therefore, leucovorin was considered more appropriate for the present mTBI repurposing framework. Future comparative studies should determine whether leucovorin and L-methylfolate exert distinct or overlapping neuroprotective effects after traumatic brain injury.

To our knowledge, no previous study has systematically evaluated the effects of LEU across behavioral, biochemical, histopathological, and immunohistochemical domains within a single experimental model of mTBI. Accordingly, the present study employed an integrated experimental approach to determine whether LEU modulates oxidative balance (TAS, TOS, OSI), neuroinflammatory signaling (TNF-α, COX-2), apoptosis-related signaling (caspase-3), neuroplasticity (BDNF), and cholinergic function (AChE) following mTBI. These molecular and tissue-level outcomes were evaluated together with functional endpoints encompassing anxiety-like and depressive-like behaviors, as well as learning and memory performance, supported by histopathological and immunohistochemical analyses.

## 2. Results

### 2.1. General Toxicity and Body Weight Changes

No mortality, unexpected adverse events, or exclusions of animals/data points occurred during the study. General health status was comparable among groups, and body weights at baseline and euthanasia did not differ significantly among the CON, mTBI, and mTBI + LEU groups (one-way ANOVA, *p* > 0.05).

After sham surgery or mTBI induction, all animals were monitored during immediate post-anesthetic recovery based on spontaneous respiration, return of the righting reflex, spontaneous movement, and absence of prolonged apnea, seizure-like activity, or delayed awakening. All animals recovered uneventfully. The mean time to recovery of the righting reflex was 18.4 ± 3.1 min in the CON group, 19.7 ± 3.8 min in the mTBI group, and 18.9 ± 3.5 min in the LT group, with no significant difference among groups (one-way ANOVA, *p* > 0.05). No animal was excluded because of abnormal post-anesthetic recovery.

### 2.2. Behavioral Tests

#### 2.2.1. mTBI Did Not Alter Locomotor Activity

**Figure 1:**
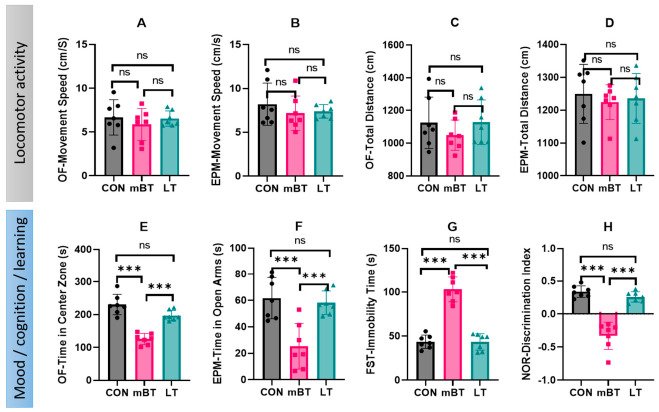
Effects of mBT and LEU treatment on behavioral performance. Anxiety-like, depression-like, locomotor, and cognitive behaviors were evaluated using standardized behavioral paradigms. (**A**) Movement speed in the OF test (cm/s). (**B**) Movement speed in the EPM (cm/s). (**C**) Total distance traveled in the open field (OF) test (cm). (**D**) Total distance traveled in the EPM test (cm). (**E**) Time spent in the central zone in the OF test (s). (**F**) Time spent in the open arms of the elevated plus maze (EPM) (s). (**G**) Immobility time in the forced swim test (FST) (s). (**H**) Discrimination index in the novel object recognition (NOR) test. Data are presented as mean ± SD. Statistical analyses were performed using one-way ANOVA followed by Tukey’s post hoc test. Dots, squares, and triangles represent individual data points corresponding to each test/group. ns, not significant; *** *p* < 0.001.

Basal locomotor activity was assessed using total distance traveled and mean movement speed in the OF and EPM paradigms. Neither mTBI induction nor LEU treatment significantly affected these parameters, with no significant differences observed among the CON, mTBI, and LT groups (all *p* > 0.05; Figure 1A–D). Although a formal neurological severity score was not performed, these locomotor measures provided an indirect assessment of gross motor function. The absence of significant group differences suggests that the observed anxiety-like, depressive-like, and recognition memory alterations were unlikely to be primarily driven by gross locomotor impairment, hypoactivity, or sedation.

#### 2.2.2. Effect of Leucovorin on Anxiety-Like Behaviors After mTBI

In anxiety-related behavioral assays, mTBI significantly reduced the time spent in the open arms of the EPM and the center zone of the OF compared with the CON group (both *p* < 0.001), indicating increased anxiety-like behavior (Figure 1E,F). LEU treatment significantly increased the time spent in the open arms of the EPM and the center zone of the OF compared with the mTBI group (both *p* < 0.001). These parameters did not differ significantly between the LT and CON groups (*p* > 0.05; Figure 1E,F).

#### 2.2.3. Effect of Leucovorin on Depression-like Behavior After mTBI

In the forced swim test, mTBI significantly increased immobility time compared with the CON group (*p* < 0.001), indicating enhanced depressive-like behavior (Figure 1G). LEU treatment significantly reduced immobility time compared with the mTBI group (*p* < 0.001), with values comparable to those of the CON group (*p* > 0.05; Figure 1G).

#### 2.2.4. Effect of Leucovorin on Recognition Memory Performance After mTBI

In the NOR test, trauma significantly reduced the discrimination index compared with the CON group (*p* < 0.001), indicating impaired recognition memory (Figure 1H). LEU treatment significantly improved discrimination performance relative to the mBT group (*p* < 0.001), restoring values to levels not significantly different from the CON group (*p* > 0.05; Figure 1H).

Collectively, behavioral outcomes across the OF, EPM, FST, and NOR tests indicate that leucovorin attenuated anxiety-like, depressive-like, and cognition-related deficits without affecting locomotor performance, supporting improvements in affective- and cognitive-related behavioral outcomes (Table 1).

Summary of behavioral test results showing the effects of mild brain trauma and leucovorin treatment on anxiety-like behavior, depressive-like behavior, cognitive performance, and locomotor activity. Primary outcome measures from the OF, EPM, FST, and NOR tests are presented together with their neurobiological interpretations. Mild brain trauma induced anxiety-like and depressive-like behavioral alterations and impaired recognition memory without significantly affecting locomotor activity. Leucovorin treatment improved these behavioral alterations, while locomotor measures remained unchanged, suggesting that the observed improvements were not primarily attributable to nonspecific motor stimulation.

### 2.3. Biochemical Evaluation

#### 2.3.1. Effect of mTBI on Biochemical Parameters

**Figure 2:**
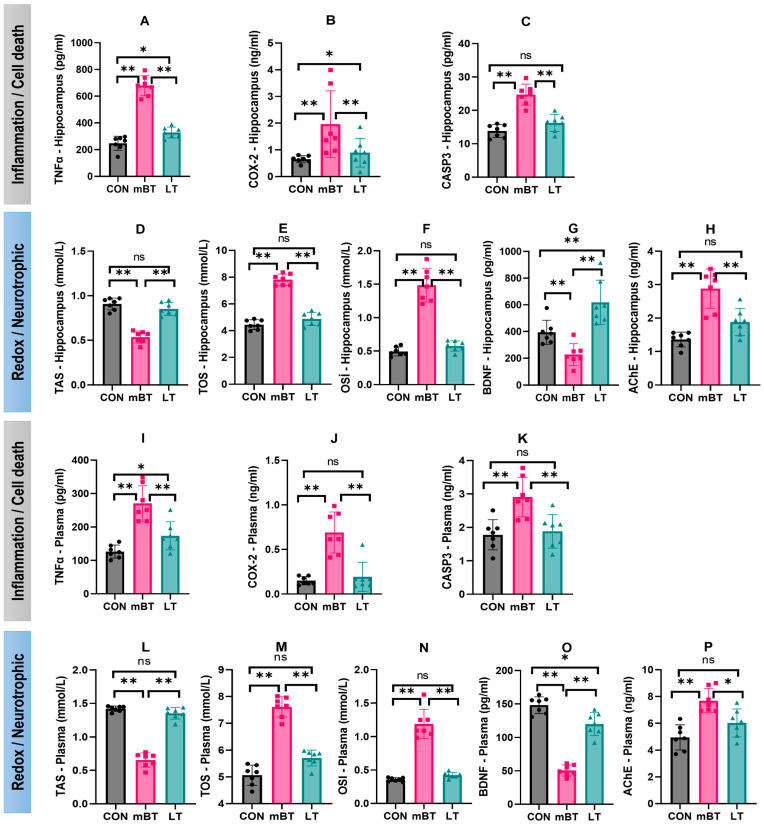
ELISA-based assessment of inflammatory, oxidative stress, apoptotic, and neurotrophic markers in hippocampal tissue and plasma. Rows 1–2 (Hippocampus): Quantification of TNF-α (**A**), COX-2 (**B**), TAS (**C**), TOS (**D**), OSI (**E**), caspase-3 (**F**), BDNF (**G**), AChE (**H**). Rows 3–4 (Plasma): Levels of TNF-α (**I**), COX-2 (**J**), TAS (**K**), TOS (**L**), OSI (**M**), caspase-3 (**N**), BDNF (**O**), and AChE (**P**). Experimental groups include CON, mBT, and LT. Data are presented as mean ± SEM, with individual data points shown to illustrate within-group variability. Data are presented as mean ± SD. Individual markers, including dots, squares, and triangles, represent individual animal values within the corresponding groups. Colors indicate the respective experimental groups. ns, not significant; * *p* < 0.05, ** *p* < 0.01.

Compared with the CON group, the mBT group exhibited marked biochemical alterations in both hippocampal tissue and plasma (Figure 2). In the hippocampus, trauma significantly increased tumor necrosis factor-α (TNF-α; *p* < 0.001), cyclooxygenase-2 (COX-2; *p* < 0.05), and caspase-3 levels (*p* < 0.001) (Figure 2A–C). Conversely, total antioxidant status (TAS) was significantly decreased (*p* < 0.001), whereas total oxidant status (TOS) and oxidative stress index (OSI) were significantly increased (both *p* < 0.001) (Figure 2D–F). In addition, hippocampal brain-derived neurotrophic factor (BDNF) levels were significantly reduced (*p* < 0.001), while acetylcholinesterase (AChE) activity was significantly increased (*p* < 0.001) (Figure 2G,H).

Parallel alterations were observed in plasma samples. The mBT group showed significant increases in plasma TNF-α and COX-2 levels (both *p* < 0.001) and caspase-3 activity (*p* < 0.01) compared with the CON group (Figure 2I–K). Plasma TAS levels were significantly reduced (*p* < 0.001), whereas TOS and OSI were significantly elevated (both *p* < 0.001) (Figure 2L–N). Plasma BDNF concentrations were significantly decreased (*p* < 0.001), accompanied by a significant increase in AChE activity (*p* < 0.001) (Figure 2O,P).

#### 2.3.2. Effect of Leucovorin on Biochemical Parameters in Rats with mTBI

LEU treatment significantly attenuated mBT-induced biochemical alterations in both hippocampal tissue and plasma (Figure 2). In the hippocampus, LEU significantly reduced TNF-α, COX-2, and caspase-3 levels compared with the mBT group (all *p* < 0.05), and these parameters did not differ significantly from those of the CON group (*p* > 0.05; Figure 2A–C). LEU also significantly increased TAS and reduced TOS and OSI compared with the mBT group (all *p* < 0.001), with redox parameters reaching values not significantly different from those of the CON group (*p* > 0.05; Figure 2D–F). Furthermore, LEU significantly increased hippocampal BDNF levels and reduced AChE activity compared with the mBT group (both *p* < 0.001; Figure 2G,H).

Plasma biochemical profiles showed a similar pattern. Plasma TNF-α, COX-2, and caspase-3 levels were significantly reduced in the LT group compared with the mBT group (all *p* < 0.01) and did not differ significantly from CON group values (*p* > 0.05; Figure 2I–K). Plasma TAS levels were significantly increased, whereas TOS and OSI were significantly decreased following LEU treatment compared with the mBT group (all *p* < 0.001; Figure 2L–N). In addition, LEU significantly increased plasma BDNF concentrations (*p* < 0.001) and reduced AChE activity (*p* < 0.01) compared with the mBT group (Figure 2O,P).

To facilitate interpretation, the measured biochemical parameters were grouped according to their involvement in distinct secondary injury domains, including neuroinflammation, oxidative stress, apoptosis-related signaling, neuroplasticity, and cholinergic dysfunction (Table 2).

The table summarizes the molecular markers used to evaluate key secondary injury domains in the mBT model, including neuroinflammation, oxidative stress/redox imbalance, apoptosis-related signaling, neuroplasticity, and cholinergic dysfunction. TNF-α and COX-2 reflect inflammatory signaling; TAS, TOS, and OSI characterize redox balance; caspase-3 reflects apoptosis-related signaling; BDNF represents neurotrophic support and synaptic plasticity-related processes; and AChE activity serves as an index of cholinergic neurotransmission relevant to cognitive and affective outcomes. Together, these biomarkers provide an integrated biochemical framework for assessing secondary injury mechanisms and the potential neuroprotective effects of leucovorin.

### 2.4. Histopathological and Immunohistochemical Examinations

#### 2.4.1. Histopathological Examinations

**Figure 3:**
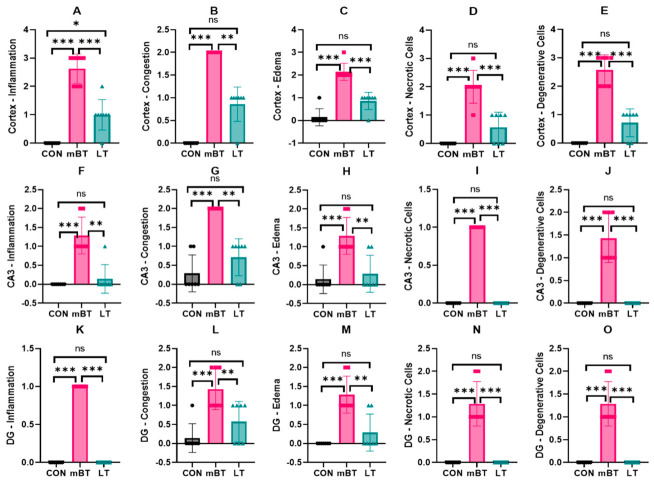
Semi-quantitative histopathological scoring of cortical and hippocampal regions. Histopathological alterations were evaluated in the cortex, CA3, and dentate gyrus (DG) regions using a semi-quantitative scoring system. Panels (**A**–**E**) represent pathological scores in the cortex: (**A**) inflammation, (**B**) congestion, (**C**) edema, (**D**) necrotic cells, and (**E**) degenerative cells. Panels (**F**–**J**) show the corresponding parameters in the CA3 region: (**F**) inflammation, (**G**) congestion, (**H**) edema, (**I**) necrotic cells, and (**J**) degenerative cells. Panels (**K**–**O**) depict the same pathological features in the dentate gyrus (DG): (**K**) inflammation, (**L**) congestion, (**M**) edema, (**N**) necrotic cells, and (**O**) degenerative cells. Data are presented as mean ± SD. Dots, squares, and triangles represent individual animal values within the corresponding groups. Colors indicate the respective experimental groups. ns, not significant; * *p* < 0.05, ** *p* < 0.01, *** *p* < 0.001.

**Figure 4:**
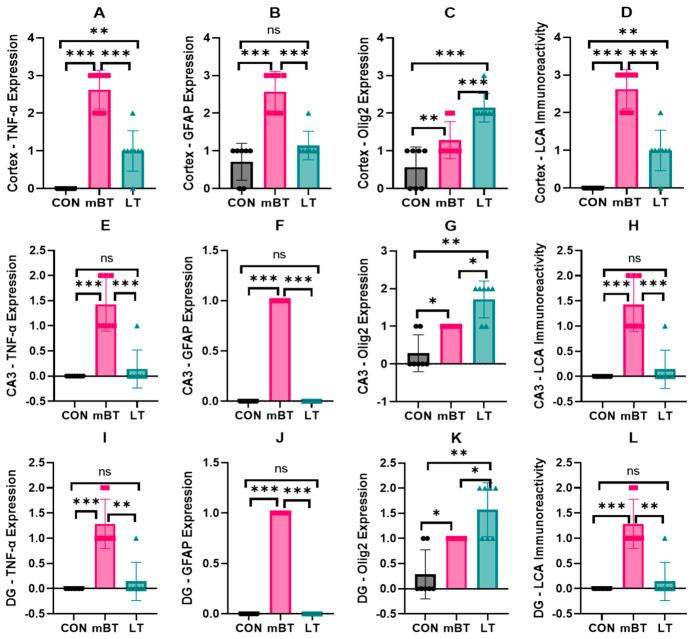
Immunohistochemical scoring of cortical and hippocampal regions. Semi-quantitative immunohistochemical analysis was performed in the cortex, CA3, and dentate gyrus (DG) regions. Panels (**A–D**) show immunoreactivity in the cortex: (**A**) TNF-α, (**B**) GFAP, (**C**) Olig2, and (**D**) LCA. Panels (**E–H**) depict the corresponding markers in the CA3 region: (**E**) TNF-α, (**F**) GFAP, (**G**) Olig2, and (**H**) LCA. Panels (**I–L**) represent immunohistochemical scoring in the dentate gyrus: (**I**) TNF-α, (**J**) GFAP, (**K**) Olig2, and (**L**) LCA. Data are presented as mean ± SD. Dots, squares, and triangles represent individual animal values within the corresponding groups. Colors indicate the respective experimental groups. ns, not significant; * *p* < 0.05, ** *p* < 0.01, *** *p* < 0.001.

**Figure 5:**
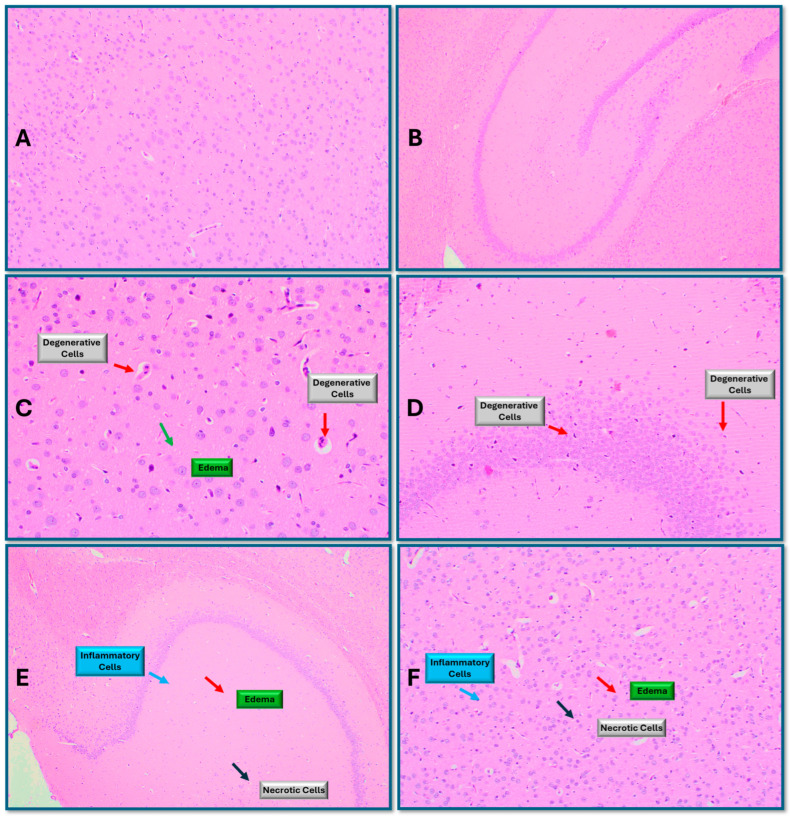
Representative histopathological images of the cortex and CA3 region: (**A**) ×4 magnification showing normal cortical architecture in the CON group; (**B**) ×4 magnification showing normal histological appearance of the CA3 region in the CON group; (**C**) ×10 magnification demonstrating degenerative neurons (red arrow) and tissue edema (green arrow) in the cortex of the mBT group; (**D**) ×4 magnification illustrating degenerative neuronal changes (red arrows) in the CA3 region of the mBT group; (**E**) ×4 magnification showing reduced edema (red arrow), inflammatory cell infiltration (blue arrow), and necrotic cells (black arrow) in the CA3 region of the LT group compared with the trauma group; and (**F**) ×10 magnification showing attenuated edema (red arrow), inflammatory cell infiltration (blue arrow), and necrotic changes (black arrow) in the cortical region of the LT group compared with the trauma group.

Histopathological scores differed significantly among groups (Kruskal–Wallis test, *p* < 0.001). The mBT group showed marked pathological alterations in the cortex, CA3, and dentate gyrus, including inflammation, vascular congestion, edema, neuronal degeneration, and necrotic changes compared with the CON group (all *p* < 0.001; Figure 3, Figure 4 and Figure 5). The greatest pathological severity was observed in the cortex, followed by the CA3 region and then the dentate gyrus. LEU treatment significantly reduced pathology scores in the cortex (*p* < 0.001 vs. mBT) and in the CA3 and dentate gyrus regions (both *p* < 0.01 vs. mBT). Pathology scores in the hippocampal subregions of LT animals did not differ significantly from those of the CON group (*p* > 0.05; Figure 4 and Figure 5).

#### 2.4.2. Immunohistochemical Examinations

**Figure 6:**
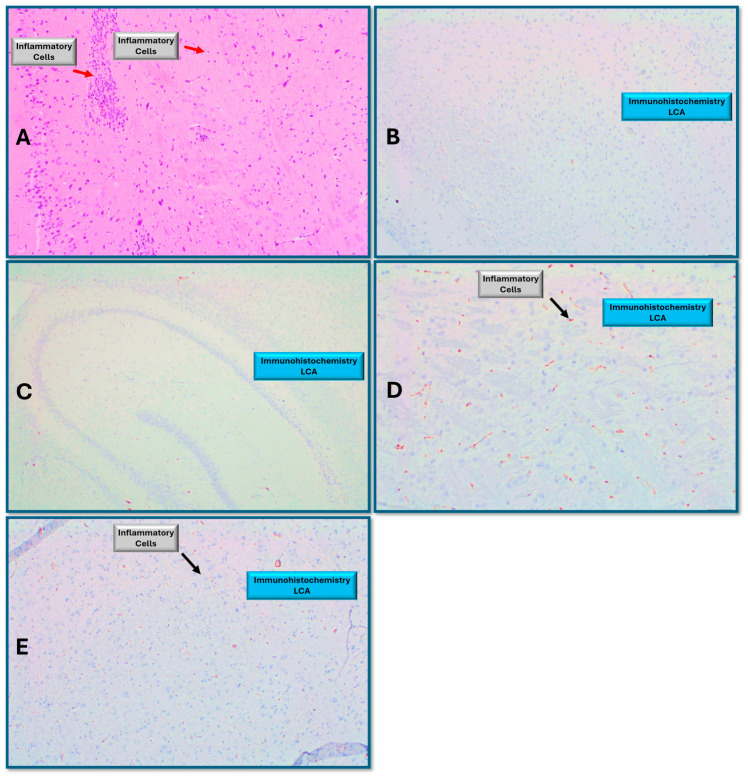
Detection of inflammatory cells in the cortex and CA3 region by H&E and LCA immunohistochemistry: (**A**) ×4 magnification showing inflammatory cell infiltration (red arrows) in the cortex of the mBT group (H&E staining); (**B**) ×4 magnification demonstrating the absence of inflammatory cell immunoreactivity in the cortical region of the CON group (LCA immunohistochemistry; negative staining); (**C**) ×4 magnification demonstrating the absence of inflammatory cell immunoreactivity in the CA3 region of the control group (LCA immunohistochemistry; negative staining); (**D**) ×10 magnification showing prominent inflammatory cell immunoreactivity (black arrow) in the mBT group, visualized by LCA immunohistochemistry; and (**E**) ×4 magnification showing reduced inflammatory cell immunoreactivity (black arrow) in the LT group compared with the trauma group (LCA immunohistochemistry).

**Figure 7:**
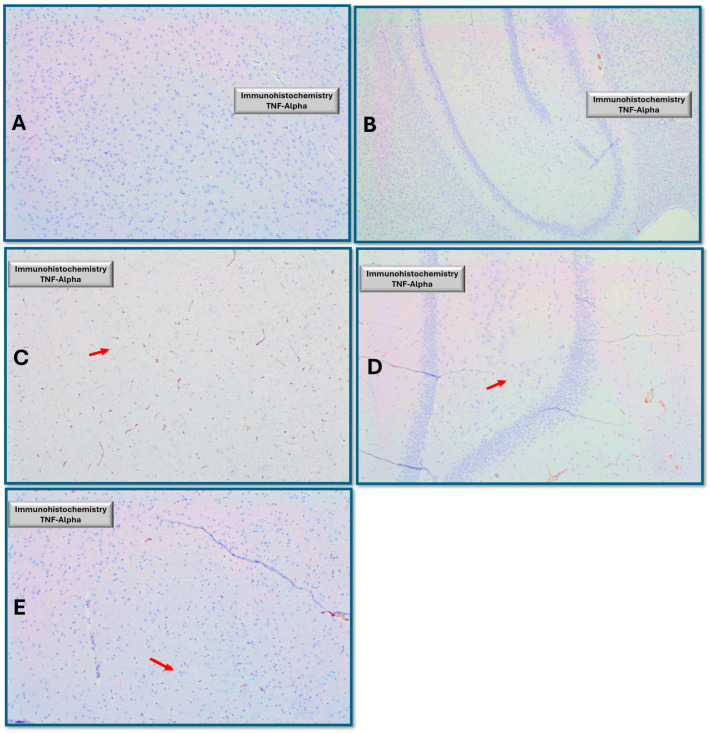
TNF-α immunohistochemical detection of inflammatory cells in the cortex and CA3 region: (**A**) ×10 magnification showing absence of TNF-α immunoreactivity in the cortical region of the CON group; (**B**) ×10 magnification showing absence of TNF-α immunoreactivity in the CA3 region of the CON group; (**C**) ×10 magnification demonstrating prominent TNF-α–positive inflammatory cells (red arrow) in the cortex of the mBT group; (**D**) ×4 magnification demonstrating TNF-α–positive inflammatory cells (red arrow) in the CA3 region of the mBT group; and (**E**) ×10 magnification showing reduced TNF-α immunoreactivity (red arrow), with sparse and mildly positive cells in the cortex of the LT group compared with the mBT group.

**Figure 8:**
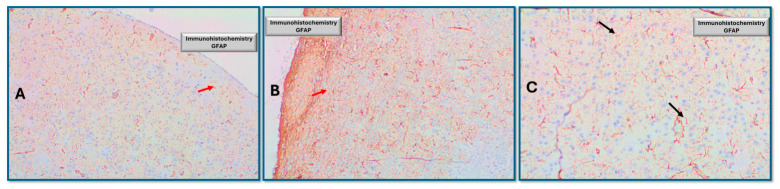
GFAP immunohistochemical evaluation of glial activation in the cortex and CA3 region: (**A**) ×10 magnification showing mild GFAP immunoreactivity in glial cells (red arrow) of the CON group; (**B**) ×10 magnification showing marked GFAP immunoreactivity indicative of reactive gliosis in the mBT group (red arrow); and (**C**) ×20 magnification showing attenuated GFAP immunoreactivity (black arrows) in glial cells of the LT group compared with the mBT group.

**Figure 9:**
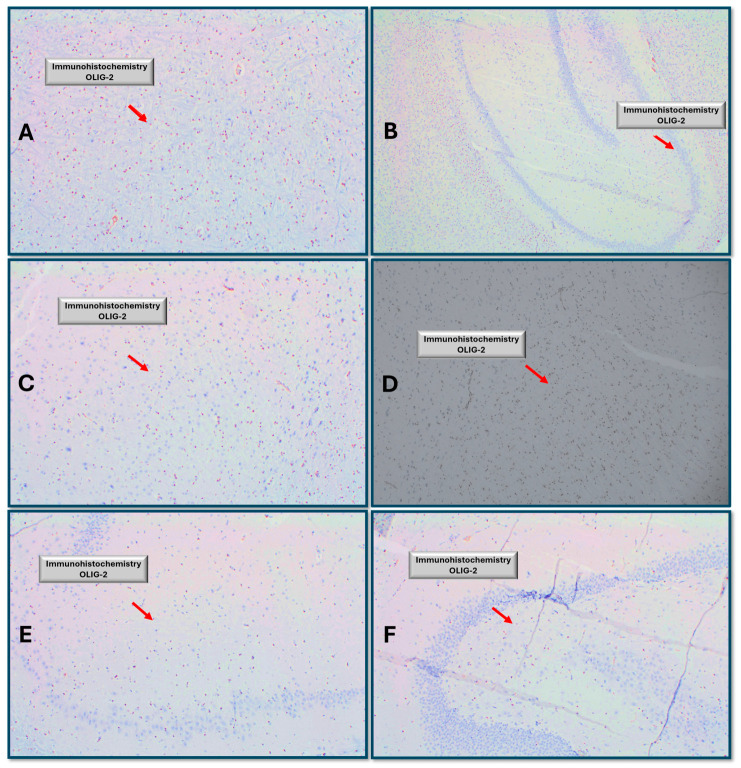
OLIG2 immunohistochemical evaluation of oligodendroglial cells in the cortex and CA3 region: (**A**) ×10 magnification showing OLIG2-positive oligodendrocytes in the cortical region (red arrow) of the CON group; (**B**) ×10 magnification showing sparsely distributed, mildly OLIG2-positive oligodendrocytes (red arrow) in the CA3 region of the CON group compared with the surrounding cortical tissue; (**C**) ×10 magnification showing moderately increased OLIG2 immunoreactivity in oligodendrocytes (red arrow) within the cortical region of the mBT group; (**D**) ×10 magnification showing marked OLIG2 immunoreactivity in oligodendrocytes (red arrow) in the cortex of the LT group; (**E**) ×20 magnification showing mildly OLIG2-positive oligodendrocytes (red arrow) in the CA3 region of the mBT group; and (**F**) ×10 magnification showing moderately increased OLIG2 immunoreactivity (red arrow) in oligodendrocytes in the CA3 region of the LT group.

Immunohistochemical analyses revealed significant region-specific alterations following trauma induction (Figure 4, Figure 6, Figure 7, Figure 8 and Figure 9). The mBT group showed markedly increased TNF-α, GFAP, and leukocyte common antigen (LCA/CD45) immunoreactivity in the cortex, CA3, and dentate gyrus compared with the CON group (all *p* < 0.001), with the highest immunoreactivity observed in the cortex, followed by the CA3 region and then the dentate gyrus (Figure 6, Figure 7 and Figure 8). LEU treatment significantly reduced TNF-α, GFAP, and LCA/CD45 immunoreactivity in the cortex (*p* < 0.001 vs. mBT) and in the CA3 and dentate gyrus regions (both *p* < 0.01 vs. mBT). Hippocampal staining levels in LT animals did not differ significantly from those of the CON group (*p* > 0.05). Following trauma induction, Olig2 immunoreactivity was significantly increased in the cortex (*p* < 0.01 vs. CON) and in the CA3 and dentate gyrus regions (both *p* < 0.05 vs. CON) (Figure 9). LEU treatment further increased Olig2 immunoreactivity across all examined regions compared with the mBT group (*p* < 0.001 in the cortex; *p* < 0.01 in the CA3 and dentate gyrus), and these values remained significantly higher than those of the CON group (*p* < 0.01).

## 3. Discussion

mTBI represents a major global public health challenge because of its high incidence and substantial long-term neurobehavioral burden. Global estimates suggest that approximately 64–74 million individuals sustain a TBI each year. Consistent with this burden, Global Burden of Disease analyses across 204 countries reported approximately 20.84 million new incident cases and 37.93 million prevalent cases in 2021, indicating that nearly 38 million individuals were living with ongoing TBI-related conditions at that time [3,35]. Mild TBI accounts for the vast majority of these injuries and is often managed outside hospital settings or remains unreported. Consequently, incidence estimates derived from hospital- or mortality-based registries are likely to underestimate the true burden of mTBI, limiting recognition of its clinical and socioeconomic impact and the contribution of secondary injury mechanisms to persistent [4,7]. Despite its classification as “mild,” mTBI is increasingly recognized as a condition that may involve persistent cognitive and affective dysfunction. These long-term alterations are thought to be driven, at least in part, by delayed secondary injury cascades involving oxidative stress, sustained neuroinflammation, metabolic dysregulation, and apoptosis-related signaling, which together compromise synaptic integrity and adaptive plasticity [10,16,36,37]. Because these secondary processes evolve over hours to days, they may create a therapeutic window for interventions that modulate upstream metabolic and inflammatory pathways rather than targeting isolated downstream events.

One biological system with emerging relevance to secondary injury biology is folate-dependent one-carbon metabolism, which integrates redox regulation, inflammatory signaling, apoptosis-related pathways, and epigenetic control of gene expression [5,17,18,19]. LEU is a bioactive reduced folate with an established clinical safety profile that bypasses dihydrofolate reductase and supports one-carbon metabolic flux. Although best known for its use in methotrexate rescue therapy, folate-dependent pathways have been increasingly implicated in redox homeostasis, immune regulation, and neuronal survival after brain injury [38]. Experimental disruption of folate metabolism exacerbates oxidative stress and caspase-dependent apoptosis, whereas restoration of reduced folate availability attenuates lipid peroxidation and limits apoptosis-related signaling [22,27,38]. One-carbon metabolism also intersects with neuroplasticity through methylation-sensitive regulation of activity-dependent genes, including brain-derived neurotrophic factor (BDNF), although any influence of LEU on neurotrophin signaling is likely to be indirect rather than trophic in nature [39,40,41,42].

The integrated behavioral profile summarized in Table 1 supports the interpretation that leucovorin-associated improvements were not attributable to nonspecific behavioral activation. At the behavioral level, the present study demonstrates that mTBI induces robust anxiety-like and depressive-like behaviors alongside impaired recognition memory, as evidenced by reduced open-arm exploration in the EPM, decreased center-zone exploration in the OF, increased immobility in the FST, and impaired NOR performance, without altering basal locomotor activity (Table 1). Preservation of total distance traveled and movement speed across behavioral paradigms supports the interpretation that reduced exploratory behavior primarily reflects affective-related alterations rather than nonspecific motor or arousal effects, consistent with prior mTBI literature [43,44]. Consistent with a metabolic-rescue framework, high-dose LEU reverses methotrexate-induced neurobehavioral toxicity in rats, demonstrating that folinic acid can restore CNS functional outputs when secondary metabolic injury is present [43]. Consistent with a metabolic-rescue framework, high-dose LEU reverses methotrexate-induced neurobehavioral toxicity in rats, demonstrating that folinic acid can restore CNS functional outputs when secondary metabolic injury is present [45]. More extensive evidence comes from folic acid and broader folate-pathway modulation studies. Folic acid has been shown to attenuate stress-induced depressive-like behavior and hippocampal redox imbalance across restraint stress, corticosterone exposure, and chronic unpredictable mild stress paradigms, typically without locomotor confounds [44,46,47,48,49]. Beyond stress models, folic acid prevents ouabain-induced hyperlocomotion and oxidative injury, linking folate status to behavioral regulation under bioenergetic challenge [50]. Although cognitive effects appear context-dependent, they have been repeatedly observed in aging-associated vulnerability, where folic acid improves memory performance alongside antioxidant status in the aged rat brain [51]. In addition, experimental disruption of folate transport through folate receptor-α antibodies produces marked affective and cognitive abnormalities, whereas preventive folinic acid-based intervention preserves behavioral function, underscoring the importance of intact folate biology for normal affective and cognitive regulation [52,53].

The domain-based organization of biomarkers (Table 2) supports the interpretation that LEU modulated coordinated inflammatory, oxidative, apoptosis-related, and plasticity-related pathways rather than producing isolated molecular effects. In the present dataset, behavioral improvements co-occurred with attenuation of TNF-α/COX-2 signaling, restoration of TAS/TOS/OSI balance, reduction in caspase-3 levels, and improvement of BDNF/AChE profiles. This pattern supports a coordinated modulation of secondary injury biology rather than isolated symptomatic effects.

At the molecular level, LEU modulated key inflammatory mediators involved in secondary injury. Tumor necrosis factor-α (TNF-α), among the earliest cytokines induced after TBI, is rapidly upregulated in the hippocampus and cortex, reflecting microglial activation and innate immune signaling [54,55,56,57]. Sustained TNF-α signaling amplifies inflammatory cascades, disrupts blood–brain barrier integrity, alters glutamatergic homeostasis, and promotes apoptosis-related cell death through NF-κB- and caspase-dependent pathways, thereby exacerbating neuronal loss and cognitive dysfunction [58,59]. Attenuation of TNF-α signaling reduces lesion severity and improves functional outcomes in experimental TBI [55,60]. In parallel, cyclooxygenase-2 (COX-2) functions as an inducible inflammatory amplifier linking arachidonic acid metabolism to prostanoid production and reactive oxygen species generation. COX-2 is rapidly and persistently upregulated in vulnerable brain regions following injury, and pharmacological inhibition reduces microglial activation, apoptosis-related signaling, and cognitive impairment [61,62,63,64]. Peripheral inflammatory readouts, including plasma TNF-α and COX-pathway metabolites, further reflect systemic immune activation and peripheral–central inflammatory crosstalk after mTBI [65,66].

In parallel with inflammatory activation, oxidative stress represents a central downstream component of secondary injury, characterized by reduced total antioxidant status (TAS), increased total oxidant status (TOS), and elevation of the oxidative stress index (OSI), particularly during the acute and early subacute phases [67]. Even modest biomechanical loading can induce mitochondrial dysfunction and neurovascular inflammation, resulting in excessive reactive oxygen and nitrogen species production that overwhelms endogenous antioxidant defenses [68,69,70]. Accordingly, TAS, TOS, and OSI serve as integrated indices of redox homeostasis rather than isolated enzymatic changes [21]. Interventions that dampen inflammatory–oxidative amplification may partially restore these indices and improve outcomes after TBI, including mTBI [71].

Downstream of inflammatory and oxidative stress, caspase-3 represents a key marker of apoptosis-related signaling. Experimental studies have demonstrated early, region-specific caspase-3 activation in hippocampal and cortical regions following TBI, reflecting convergence of mitochondrial dysfunction, excitotoxicity, and inflammatory signaling [72]. Pharmacological inhibition of caspase pathways reduces neuronal apoptosis and improves functional recovery, supporting an important contribution of caspase-dependent signaling to secondary injury progression [60]. Plasma caspase-3, by contrast, likely reflects systemic apoptotic and inflammatory turnover rather than a direct index of central neuronal death and should therefore be interpreted cautiously [73].

Plasticity-related systems are also disrupted following mTBI. Hippocampal BDNF is reduced in a time- and region-dependent manner even after injuries classified as mild, linking impaired neurotrophin signaling to synaptic dysfunction and cognitive deficits [74]. Although heterogeneous recovery patterns have been reported depending on injury severity and timing, restoration of BDNF often parallels cognitive improvement when secondary inflammatory and oxidative cascades are attenuated [74,75]. Cholinergic signaling is similarly vulnerable; acetylcholinesterase (AChE) shows region- and time-dependent alterations after TBI, with hippocampal changes linked to cognitive dysfunction and plasma changes potentially reflecting systemic inflammatory stress [76,77].

Histopathological and immunohistochemical analyses provide integrative tissue-level support for these molecular processes. mTBI produced prominent neuroinflammation, microvascular congestion, edema, and neuronal degeneration (Figure 3). These findings are consistent with delayed secondary injury biology rather than merely transient mechanical disruption [2,67]. LEU treatment significantly reduced these pathological indices, suggesting relative preservation of tissue integrity and stabilization of the neurovascular microenvironment. Such tissue-level protection aligns with preclinical mTBI literature demonstrating that interventions targeting oxidative–inflammatory amplification can reduce histopathological damage after weight-drop injury [78]. Notably, in a closely related rat mTBI model, dexpanthenol reduced oxidative injury markers and cortical neuronal damage, reinforcing the concept that limiting secondary oxidative and apoptosis-related cascades can translate into improved histological outcomes [79].

Immunohistochemically, mTBI elicited region-dependent increases in inflammatory and glial markers across the cortex, CA3, and dentate gyrus, with pronounced upregulation of TNF-α, GFAP, and LCA immunoreactivity reflecting inflammatory signaling, astrocytic reactivity, and leukocyte-associated immune activation [9,57]. Greater cortical vulnerability likely reflects direct mechanical and vascular exposure, whereas graded involvement of hippocampal subfields is consistent with known differences in excitotoxic and inflammatory susceptibility [80,81]. LEU markedly attenuated TNF-α, GFAP, and LCA immunoreactivity, suggesting reduced inflammatory–glial activation and relative preservation of tissue homeostasis [16,59]. In contrast, Olig2 immunoreactivity was increased following trauma and further enhanced by LEU, consistent with engagement of oligodendrocyte lineage-related responses involved in axonal support and white-matter remodeling [82]. Because Olig2 was increased beyond control levels in the LEU-treated group, this response may reflect sustained oligodendroglial repair-associated activity rather than nonspecific gliosis alone [83].

**Figure 10:**
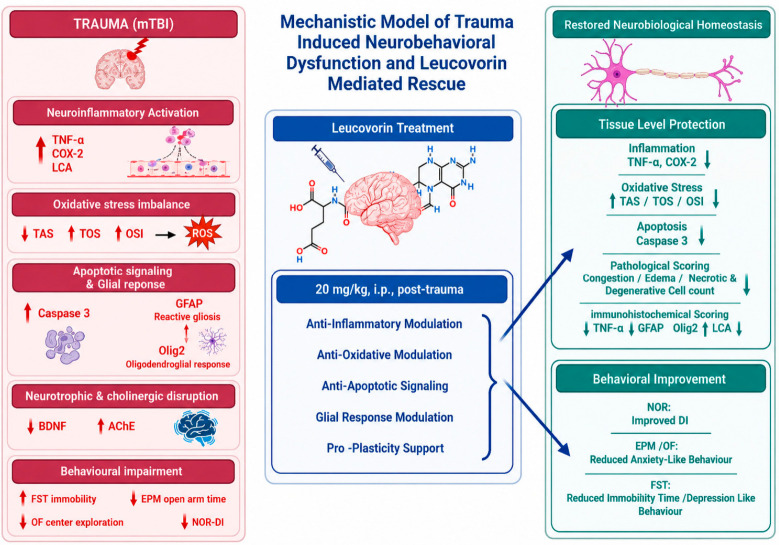
Proposed mechanistic model of trauma-induced neurobehavioral dysfunction and leucovorin-mediated neuroprotection.

Taken together, these findings support a putative secondary injury framework in which pro-inflammatory signaling (TNF-α, COX-2), oxidative stress/redox imbalance (TAS/TOS/OSI), apoptosis-related signaling (caspase-3), and disruption of plasticity- and cholinergic function-related systems (BDNF, AChE) are mechanistically interconnected. This framework, summarized in Figure 10, highlights the interplay among inflammation, redox dysregulation, apoptosis-related signaling, and neuronal vulnerability following mTBI.

Mild traumatic brain injury (mTBI) initiates a progressive secondary injury cascade characterized by mitochondrial dysfunction, excessive reactive oxygen species (ROS) production, and disruption of cellular redox homeostasis. These alterations promote astrocytic and microglial activation, thereby amplifying pro-inflammatory signaling pathways involving tumor necrosis factor-α (TNF-α) and cyclooxygenase-2 (COX-2), which may further aggravate oxidative stress and neuronal vulnerability. Sustained neuroinflammation and redox imbalance can converge on apoptosis-related pathways, particularly through caspase-3 activation, ultimately contributing to synaptic destabilization and impaired cognitive function.

Leucovorin (LEU), a reduced folate and active participant in one-carbon metabolism, is proposed to act at an upstream metabolic level by supporting methylation capacity and cellular redox balance. Through modulation of oxidative stress parameters (TAS/TOS/OSI), LEU may attenuate ROS accumulation, reduce glia-mediated inflammatory signaling, and limit the activation of apoptosis-related cascades. These combined effects may contribute to stabilization of the neuronal microenvironment, preservation of synaptic integrity, and improved behavioral outcomes. In addition, LEU may indirectly influence neuroplasticity-related pathways, including BDNF signaling, through epigenetic and metabolic mechanisms rather than direct trophic stimulation. Accordingly, the observed cognitive improvement may be interpreted as a downstream consequence of metabolic and inflammatory regulation rather than primary neurotrophic activation.

Within this context, leucovorin (LEU) may act as an upstream metabolic modulator that limits the propagation of inflammatory and oxidative amplification rather than directly targeting individual downstream mediators. By improving redox balance and attenuating glia-driven inflammatory signaling, LEU may reduce apoptosis-related burden and support synaptic and circuit-level integrity. Accordingly, the observed behavioral improvements are best interpreted as downstream consequences of metabolic and inflammatory stabilization, providing a coherent mechanistic framework for LEU-mediated neuroprotection after mTBI (Figure 10).

Limitations of the present study should be acknowledged. This study used a single-dose leucovorin regimen administered at one acute post-injury time point in a male rodent model of mTBI, which limits conclusions regarding dose–response relationships, optimal therapeutic timing, repeated-dose protocols, sex-related differences, and clinical generalizability. Molecular analyses were restricted to selected secondary injury markers, and mitochondrial markers were not directly assessed.

A formal neurological severity assessment, such as the modified neurological severity score (mNSS), rotarod, beam-walk, or sensory reflex testing, was not performed. Although OF and EPM locomotor parameters, including total distance traveled and movement speed, provided indirect evidence against gross motor impairment, these measures do not exclude subtle sensory, reflex, balance, or coordination deficits. In addition, cognition-related performance was assessed only using the NOR test; therefore, the findings should be interpreted as alterations in recognition memory rather than global cognitive impairment.

Finally, selected histological and immunohistochemical findings were not validated by Western blot analysis. Although H&E staining, semi-quantitative histopathological scoring, IHC, and ELISA-based assays provided complementary evidence of tissue injury, neuroinflammation, glial activation, apoptosis-related signaling, oxidative stress, and neurotrophic alterations, future studies should include Western blotting, qPCR, or multiplex protein-based approaches to further validate markers such as TNF-α, GFAP, LCA/CD45, Olig2, caspase-3, and BDNF.

## 4. Materials and Methods

The study protocol was approved by the KONUDAM Institutional Animal Ethics Committee (Approval No: 2024-21) and conducted in accordance with national regulations, ARRIVE 2.0 guidelines, and EU Directive 2010/63/EU, with ethical oversight guided by the Reduction principle of the 3Rs; the ARRIVE reporting guideline was used during manuscript preparation, and the completed checklist is provided as Supplementary Material [84,85].

Twenty-one healthy male Wistar albino rats (10 weeks of age; 200–300 g) were sourced from the accredited KONUDAM Experimental Medicine Application and Research Center of Necmettin Erbakan University, KONYA/TÜRKİYE. All animals were experimentally naïve at the time of inclusion. Housing conditions were standardized, with a controlled temperature (22 ± 2 °C), relative humidity (50 ± 5%), and a 12 h light/dark cycle. Animals had unrestricted access to standard chow and water.

Animals were randomly assigned to three experimental groups (*n* = 7 per group) using Random Allocation Software (RAS) version 1.0 to ensure unbiased assignment. Group size was determined using the resource equation method, an approach suitable when reliable prior effect size estimates are unavailable, and it supports experimental validity while minimizing animal use [86].

### 4.1. Experimental Design

**Figure 11:**
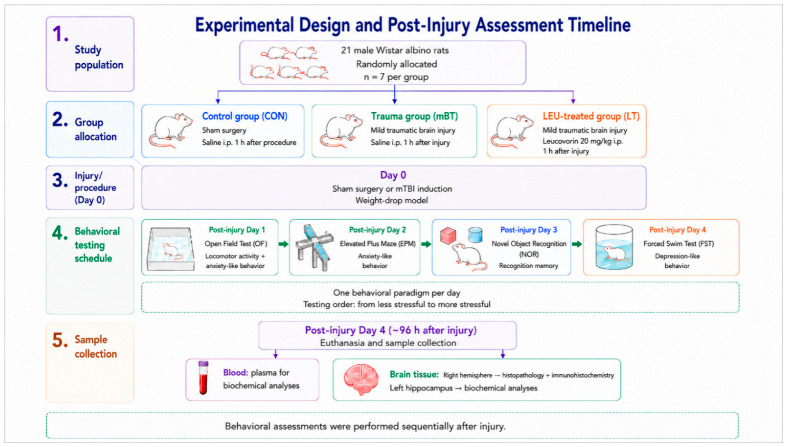
Experimental design and post-injury assessment timeline. Male Wistar albino rats were randomly allocated into control, trauma, and leucovorin-treated groups. Following sham surgery or mTBI induction on day 0, behavioral assessments were performed sequentially from post-injury day 1 to day 4 using OF, EPM, NOR, and FST paradigms. At approximately 96 h after injury, animals were euthanized, and blood and brain tissues were collected for biochemical, histopathological, and immunohistochemical analyses.

A schematic flow diagram summarizing group allocation, treatment administration, behavioral testing schedule, and sample collection is provided in Figure 11.

Study groups:

Control group (CON): Sham-operated rats underwent a midline scalp incision, and the coronal and lambdoid sutures were identified. The scalp was irrigated with sterile saline, and the incision was closed with sutures. Rats received a single intraperitoneal (IP) dose of saline 1 h after surgery (0.9% NaCl, 0.1 mL/100 g).

Trauma group (mBT): Rats underwent the mild brain trauma model. After trauma induction, rats received a single IP dose of saline 1 h after trauma (0.9% NaCl, 0.1 mL/100 g).

LEU-treated group (LT): Rats underwent the mild brain trauma model. After trauma induction, rats received a single IP dose of LEU at 20 mg/kg 1 h after trauma (Calcium Folinate™, Pfizer^®^, New York, NY, USA).

In accordance with the 3R principles and as required by the KONUDAM Ethics Committee, a treatment-only group was excluded from the study to minimize animal use. This approach is consistent with previous experimental studies in which treatment-only groups were similarly omitted [78,79,87,88,89].

Following surgery and trauma induction, LEU or saline was administered intraperitoneally as a single dose, behavioral assessments (OF, EPM, NOR, and FST) were conducted sequentially during the post-injury period, and animals were euthanized for biochemical, histopathological, and immunohistochemical analyses after completion of behavioral testing.

Leucovorin was administered 1 h after mTBI induction to evaluate its potential effect as an acute post-injury therapeutic intervention rather than as a prophylactic treatment. This time point was selected to target the early secondary injury phase, during which oxidative stress, neuroinflammatory signaling, mitochondrial dysfunction, and apoptosis-related pathways begin to evolve after the primary mechanical insult [90,91,92]. The 1 h post-injury schedule was therefore considered appropriate for modeling an early therapeutic intervention during the acute phase of mTBI and is consistent with previous experimental TBI studies evaluating neuroprotective strategies after trauma [78,79].

Although a formal protocol registration was not performed prior to study initiation, the experimental design, primary outcomes, and statistical analysis plan were defined a priori in accordance with the ARRIVE 2.0 guidelines. These elements were specified before data collection and were not modified during the study.

### 4.2. Mild Brain Trauma Model

Rats were anesthetized with an intraperitoneal injection of ketamine (50 mg/kg; Ketalar, Parke Davis, Istanbul, Türkiye) combined with xylazine (10 mg/kg; Rompun, Bayer, Leverkusen, Germany). This anesthetic regimen was selected because ketamine/xylazine provides a reliable surgical anesthetic plane and is commonly used in rodent experimental TBI and neurobehavioral studies, including weight-drop and diffuse brain injury models designed to evaluate neuroprotective interventions [78,79,93]. Although no single anesthetic protocol is universally accepted as the standard for all rodent TBI models, the same regimen was applied consistently across all experimental groups to minimize anesthesia-related variability. The selected weight-drop model has also been widely used in experimental studies investigating oxidative stress, neuroinflammation, and neuroprotective strategies after TBI [78,79].

For the injury procedure, animals were placed in the prone position on a platform covered with a 10 cm-thick foam pad to allow controlled deceleration after impact. After a midline scalp incision, the coronal and lambdoid sutures were identified, and a stainless-steel disk measuring 10 mm in diameter and 3 mm in thickness was fixed to the skull at the midline between the sutures using bone wax. This disk was used to distribute the impact force evenly, as previously described [93,94].

Impact severity was standardized using a fixed weight-drop protocol. A 300 g weight was dropped from a height of 70 cm through a vertical copper tube onto the stainless-steel disk positioned at the midline between the coronal and lambdoid sutures. Reproducible impact delivery and force distribution were ensured by maintaining constant weight, drop height, impact location, and disk dimensions across animals. Immediately after impact, the metal disk was removed, and the skull surface was visually inspected before scalp closure to confirm the absence of skull fracture. No skull fracture, marked cranial deformation, or abnormal bleeding was observed in any animal. Animals with skull fracture or gross cranial damage would have been excluded from the analysis; however, no such exclusion was required.

In the CON group, rats underwent the same surgical exposure, including scalp incision and identification of the coronal and lambdoid sutures, but no weight-drop impact was applied.

All surgical procedures and trauma induction were performed under deep general anesthesia with ketamine/xylazine, and animals were not exposed to the impact procedure while conscious. No additional opioid, non-steroidal anti-inflammatory drug, or paracetamol/acetaminophen was administered because the primary outcomes of the study included neuroinflammatory markers, oxidative stress indices, apoptosis-related signaling, glial activation, neuroplasticity-related markers, cholinergic function, and affective- and cognition-related behavioral endpoints. Analgesic agents may modify injury-related biological and behavioral readouts in experimental TBI, particularly inflammatory, oxidative, glial, and neurobehavioral outcomes [95,96,97,98]. In addition, paracetamol/acetaminophen was not used as a non-opioid alternative because it has been shown to influence CNS-relevant oxidative stress, apoptosis-related signaling, synaptic plasticity, BDNF expression, and cognitive or behavioral outcomes in experimental models [99,100,101,102,103]. Animals were closely monitored during anesthetic recovery and throughout the postoperative period for signs of pain, distress, abnormal behavior, impaired mobility, or unexpected complications. No mortality or unexpected adverse events were observed.

### 4.3. Behavioral Assessments

All behavioral evaluations were conducted in noise-isolated rooms under controlled illumination conditions (100 lux) at the KONUDAM Experimental Application and Research Center (Konya, Türkiye). Automated video tracking and analysis were performed using the EthoVision XT 11 system (Noldus, The Netherlands). Before each behavioral assessment, rats were allowed to acclimate to the testing room for 30 min. All apparatuses were cleaned with 70% ethanol between animals to eliminate olfactory cues.

Behavioral assessments were performed according to a predefined post-injury timeline designed to minimize fatigue, stress accumulation, and carry-over effects. Following sham surgery or mTBI induction on day 0, animals received saline or leucovorin 1 h after the procedure. The behavioral battery was distributed across four consecutive post-injury days, with only one paradigm performed per day and no more than 12 behavioral recordings conducted per day under standardized room conditions.

Tests were conducted from less stressful to more stressful paradigms: OF on post-injury day 1 to assess locomotor activity and anxiety-like behavior, EPM on day 2 to further evaluate anxiety-like behavior, NOR on day 3 to assess recognition memory, and FST on day 4 to evaluate depressive-like behavior. The FST was performed last because of its relatively stressful nature and potential carry-over effects. Movement speed and total distance in the OF and EPM tests were also analyzed to determine whether fatigue, hypoactivity, or gross motor suppression influenced behavioral performance. After completion of the behavioral battery, animals were euthanized, and blood and brain tissues were collected for biochemical, histopathological, and immunohistochemical analyses.

#### 4.3.1. Open Field Test (OFT)

Locomotor activity and anxiety-related behavior were assessed using the open field (OF) paradigm, as described by Öz (2024) [104]. Testing was performed in an 80 × 80 × 40 cm (length × width × height) arena, and total distance traveled, movement speed, and time spent in the center zone were recorded.

#### 4.3.2. Elevated Plus Maze (EPM)

The elevated plus maze (EPM) test was performed using a plus-shaped apparatus elevated 50 cm above the floor. The maze consisted of two opposing open arms and two opposing closed arms arranged perpendicularly around a central square platform. Each arm measured 50 × 10 cm (length × width), and the central platform measured 10 × 10 cm (length × width). The closed arms were enclosed by 40 cm high opaque walls, whereas the open arms had no side walls.

At the beginning of each trial, each rat was placed in the central square facing one of the open arms and allowed to explore the maze freely for 5 min. During the test period, time spent in the open arms and movement speed were recorded as indices of anxiety-like behavior and locomotor activity, respectively. The apparatus was cleaned with 70% ethanol between trials to eliminate olfactory cues [104].

#### 4.3.3. Novel Object Recognition (NOR)

Recognition memory was assessed using the NOR test. The procedure consisted of three phases.

Habituation: Rats freely explored the empty arena for 10 min.

Training: Two identical objects were placed in opposite corners, and animals explored for 5 min.

Testing: After a 1.5 h retention interval, one familiar object was replaced with a novel object, and exploration was recorded for 5 min.

Object exploration was defined as touching or approaching within 2 cm. The discrimination index (DI) was computed as:

DI = (Novel exploration − Familiar exploration)/(Novel exploration + Familiar exploration).

#### 4.3.4. Forced Swim Test (FST)

Depressive-like behavior was evaluated according to the protocol of Aslanlar (2024) [105]. The test was conducted in water at 25 °C for 5 min, and immobility time was recorded for analysis. Immobility time during the test session was used for analysis.

### 4.4. Tissue Collection

Sample collection was performed on post-injury day 4, after completion of the final behavioral assessment, corresponding to approximately 96 h after sham surgery or mTBI induction. Animals were anesthetized using the previously described protocol. Intracardiac blood samples (3–4 mL) were collected into EDTA tubes and centrifuged at 3200 rpm for 10 min at 4 °C. Plasma samples were stored at −80 °C until biochemical analyses. Rats were then euthanized by cervical dislocation, and brains were rapidly removed and placed on ice. Each brain was divided into right and left hemispheres. The right hemisphere was fixed in 4% paraformaldehyde and used for histopathological and immunohistochemical analyses. From the left hemisphere, hippocampal tissue was dissected on ice, homogenized in phosphate buffer (pH 7.4), and centrifuged at 4000 rpm for 10 min at 4 °C. The resulting supernatants were stored at −80 °C until biochemical analyses.

### 4.5. Biochemical Analyses

Plasma and hippocampal concentrations of BDNF, AChE, TNF-α, COX-2, and caspase-3 were measured using rat-specific ELISA kits from Elabscience^®^, Houston, TX, USA (E-EL-R1235, E-EL-R0355, E-EL-R0019, E-EL-R0792, and E-EL-R0160, respectively). Absorbance was read at 450 ± 2 nm using a BioTek ELx800 microplate reader (Winooski, VT, USA), and concentrations were calculated from standard curves. Total antioxidant status (TAS) and total oxidant status (TOS) were measured using commercial assay kits from Rel Assay^®^, Gaziantep, Türkiye (RL0017 and RL0024, respectively). The oxidative stress index (OSI) was calculated as the ratio of TOS to TAS. OSI (arbitrary units) = TOS (μmol H_2_O_2_ equivalent/L)/TAS (μmol Trolox equivalent/L) [106,107,108]. All ELISA measurements were conducted in duplicate to ensure analytical reliability.

### 4.6. Histopathological Examination

Brain tissues were fixed, embedded in paraffin, and cut into 5 µm coronal sections using a rotary microtome (Leica Biosystems, Nussloch, Germany). Hematoxylin–eosin (H&E) staining was performed for morphological evaluation of neuronal and tissue architectures. Stained sections were examined under an Olympus BX43 photomicroscope (Olympus, Tokyo, Japan).

Histopathological assessment focused on the cerebral cortex, dentate gyrus (DG), and CA3 region of the hippocampus to capture region-specific vulnerability to injury. Neuronal injury was evaluated by examining key morphological indicators of structural damage and secondary injury processes, including neuronal degeneration, cellular edema, vascular congestion, inflammatory cell infiltration, and necrotic changes. These features were interpreted as reflecting disruption of neuronal integrity, microvascular dysfunction, and neuroinflammatory activation.

Each parameter was scored using a standardized 4-point ordinal scale: 0 = absent/normal, 1 = mild involvement, defined as focal changes with low frequency, 2 = moderate involvement, defined as multifocal and readily apparent changes, and 3 = severe involvement, defined as diffuse and/or extensive changes. Scoring was performed within predefined regions of interest to ensure consistency and comparability across experimental groups.

### 4.7. Immunohistochemistry

#### 4.7.1. Immunohistochemical Staining

Immunohistochemical analyses were performed to characterize neuroinflammatory, glial, and lineage-specific cellular responses within brain tissue. Automated staining was conducted using a VENTANA Discovery XT system with RUO XT OptiView DAB IHC v4 software (Ventana Medical Systems, Inc., Tucson, AZ, USA) to ensure procedural standardization and minimize operator-dependent variability. Antigen–antibody complexes were visualized using the OptiView DAB IHC Detection Kit with signal amplification according to the manufacturer’s instructions.

Antigen retrieval was performed using Cell Conditioning Solution 2 (CC2) for 32 min to optimize epitope exposure. The Universal Linker and horseradish peroxidase (HRP) multimer were applied sequentially for 8 min each, followed by the OptiView Amplifier and Amplification Multimer for 4 min each to enhance detection sensitivity while preserving tissue morphology.

Immunolabeling was performed using antibodies against tumor necrosis factor-α (TNF-α; SP130) to assess neuroinflammatory activation, leukocyte common antigen (LCA; 2B11 + PD7/26) to evaluate immune cell infiltration, glial fibrillary acidic protein (GFAP; EP672Y) to quantify astroglial reactivity, and oligodendrocyte transcription factor-2 (Olig2; EP112) to identify oligodendroglial lineage responses. Staining was completed using the OptiView DAB IHC Detection Kit on the automated slide staining platform under standardized conditions.

Negative controls were included in each staining run by omitting the primary antibody and substituting antibody diluent to exclude nonspecific background staining. Isotype controls matched for host species and immunoglobulin class were processed in parallel to verify antibody specificity and rule out Fc-mediated or nonspecific binding. Positive control tissues with established expression of the target antigens were stained concurrently under identical conditions to confirm antibody performance and assay reliability.

#### 4.7.2. Quantification and Scoring

Immunoreactivity was evaluated using both semi-quantitative scoring and digital image analysis to improve reproducibility and reduce observer bias. For semi-quantitative analysis, staining intensity and distribution were scored using a predefined ordinal scale by two independent observers blinded to the experimental groups, and discrepancies were resolved by consensus.

For quantitative analysis, digital images were captured under standardized illumination and magnification settings. The percentage of immunopositive area (% area) and integrated optical density (IOD) were measured using Olympus cellSens software, version 4.4.1 (Olympus Corporation, Tokyo, Japan)., with threshold parameters kept constant across all sections. Quantitative values were averaged from multiple non-overlapping fields per region of interest to account for regional heterogeneity and improve representativeness. All analyses were performed in a blinded manner to minimize observer bias.

Immunohistochemical images were obtained from predefined regions of interest in the right hemisphere, including the cerebral cortex, CA3 region, and dentate gyrus. These regions were selected because they represent cortical and hippocampal areas vulnerable to secondary injury after mTBI and correspond to the same anatomical regions used for histopathological scoring. For each marker, representative images were captured from comparable regions across all experimental groups under standardized magnification, illumination, and exposure settings. Quantitative and semi-quantitative analyses were performed using multiple non-overlapping fields within each predefined region of interest.

### 4.8. Statistical Analysis

All statistical analyses were performed using IBM SPSS Statistics for Windows (version 27.0; IBM Corp., Armonk, NY, USA). Distributional assumptions were evaluated using visual methods, including histograms and Q–Q plots, together with formal normality tests, including the Kolmogorov–Smirnov and Shapiro–Wilk tests. Normally distributed data are presented as mean ± standard deviation, whereas non-normally distributed variables are reported as median (minimum–maximum). Categorical variables are expressed as frequency (n) and percentage (%).

Between-group comparisons were selected according to distributional characteristics and measurement level. Ordinal data and continuous variables that did not meet parametric assumptions were analyzed using the Kruskal–Wallis test. When a significant overall difference was detected, pairwise comparisons were performed using Dunn’s post hoc test with Bonferroni correction.

For normally distributed continuous variables, differences among independent groups were assessed using one-way analysis of variance (ANOVA). Homogeneity of variance was evaluated using Levene’s test. When the assumption of equal variances was met, Tukey’s honestly significant difference (HSD) test was applied for post hoc analysis. When variances were unequal, Welch’s ANOVA followed by the Games–Howell post hoc test was used. All tests were two-sided, and *p* < 0.05 was considered statistically significant.

## 5. Conclusions

In conclusion, the present study indicates that LEU modulates multiple components of secondary injury following mTBI. LEU treatment attenuated oxidative stress and inflammatory activation, reduced apoptosis-related signaling, and supported neurotrophic and cholinergic balance. These effects were accompanied by relative preservation of neuronal tissue architecture and improvements in recognition memory, anxiety-like behavior, and depressive-like behavior without evidence of nonspecific locomotor activation. Together, these findings suggest that LEU may function as an upstream metabolic modulator capable of limiting secondary injury cascades after mTBI. Further experimental and translational studies are warranted to define its therapeutic potential, optimal dosing strategy, and treatment window in TBI.

## Figures and Tables

**Table 1:** Behavioral outcomes and neurobiological interpretation of leucovorin (LEU) effects in the mild traumatic brain injury (mTBI) model.

Behavioral Test	Primary Outcome Measure	Effect of mTBI	Effect of Leucovorin (LEU)	Neurobiological Interpretation
Open Field (OF)	Time spent in center zone	Significant reduction	Restored to control-like levels	Reduced center exploration reflects anxiety-like behavior driven by limbic neuroinflammation and oxidative stress; normalization indicates anxiolytic-like recovery without locomotor confounds
Open Field (OF)	Total distance traveled/movement speed	No change	No change	Confirms that behavioral alterations are not due to motor impairment or sedation, supporting specificity of affective changes
Elevated Plus Maze (EPM)	Time spent in open arms	Significant reduction	Restored to control-like levels	Reduced open-arm exploration reflects heightened anxiety-like behavior associated with hippocampal–amygdalar dysfunction and inflammatory signaling
Elevated Plus Maze (EPM)	Movement speed	No change	No change	Excludes nonspecific effects on locomotion or arousal
Forced Swim Test (FST)	Immobility time	Significant increase	Normalized to control levels	Increased immobility indicates depression-like behavior, linked to inflammatory cytokines, oxidative stress, and impaired monoaminergic/neurotrophic signaling
Novel Object Recognition (NOR)	Discrimination index (DI)	Marked impairment	Restored to control-like levels	Impaired DI reflects hippocampus-dependent recognition memory deficits, associated with reduced BDNF, increased oxidative stress, and cholinergic dysregulation
Across tests	Consistency of locomotor parameters	Preserved	Preserved	Confirms that LEU-induced improvements reflect true affective and cognitive recovery, not behavioral activation or motor stimulation

**Table 2:** Biochemical markers representing major secondary injury mechanisms in mild traumatic brain injury (mTBI).

Secondary Injury Domain	Biochemical Marker	Biological Significance in mTBI
Neuroinflammation	TNF-α	Early pro-inflammatory cytokine; amplifies microglial activation, blood–brain barrier dysfunction, and neuronal injury
	COX-2	Inducible inflammatory enzyme; promotes prostanoid synthesis, oxidative stress, and delayed neuronal damage
Oxidative stress/redox imbalance	TAS (Total Antioxidant Status)	Reflects overall antioxidant capacity and endogenous defense against reactive oxygen species
	TOS (Total Oxidant Status)	Indicates cumulative oxidant burden and reactive oxygen species production
	OSI (Oxidative Stress Index)	Integrated indicator of redox imbalance calculated as TOS/TAS; reflects net oxidative stress severity
Apoptosis	Caspase-3	Executioner caspase mediating programmed neuronal cell death during secondary injury
Neuroplasticity/trophic support	BDNF	Regulates synaptic plasticity, neuronal survival, and learning–memory processes
Cholinergic dysfunction	AChE	Controls acetylcholine availability; dysregulation contributes to cognitive and affective impairment

## Data Availability

The original contributions presented in this study are included in the article/Supplementary Material. Further inquiries can be directed to the corresponding author.

## Supplementary Materials

The following supporting information can be downloaded at: https://www.mdpi.com/article/10.3390/ph19060865/s1. The ARRIVE reporting checklist.

## References

[1] Cassidy J.D., Carroll L., Peloso P., Borg J., Von Holst H., Holm L., Kraus J., Coronado V. (2004). Incidence, risk factors and prevention of mild traumatic brain injury: Results of the WHO Collaborating Centre Task Force on Mild Traumatic Brain Injury. J. Rehabil. Med..

[2] Maas A.I., Menon D.K., Adelson P.D., Andelic N., Bell M.J., Belli A., Bragge P., Brazinova A., Büki A., Chesnut R.M. (2017). Traumatic brain injury: Integrated approaches to improve prevention, clinical care, and research. Lancet Neurol..

[3] Dewan M.C., Rattani A., Gupta S., Baticulon R.E., Hung Y.-C., Punchak M., Agrawal A., Adeleye A.O., Shrime M.G., Rubiano A.M. (2018). Estimating the global incidence of traumatic brain injury. J. Neurosurg..

[4] Carroll L.J., Cassidy J.D., Cancelliere C., Côté P., Hincapié C.A., Kristman V.L., Holm L.W., Borg J., Nygren-de Boussard C., Hartvigsen J. (2014). Systematic review of the prognosis after mild traumatic brain injury in adults: Cognitive, psychiatric, and mortality outcomes: Results of the International Collaboration on Mild Traumatic Brain Injury Prognosis. Arch. Phys. Med. Rehabil..

[5] Rabinowitz A.R., Levin H.S. (2014). Cognitive sequelae of traumatic brain injury. Psychiatr. Clin..

[6] Levin H.S., Diaz-Arrastia R.R. (2015). Diagnosis, prognosis, and clinical management of mild traumatic brain injury. Lancet Neurol..

[7] McInnes K., Friesen C.L., MacKenzie D.E., Westwood D.A., Boe S.G. (2017). Mild Traumatic Brain Injury (mTBI) and chronic cognitive impairment: A scoping review. PLoS ONE.

[8] Werner C., Engelhard K. (2007). Pathophysiology of traumatic brain injury. Br. J. Anaesth..

[9] Loane D.J., Kumar A. (2016). Microglia in the TBI brain: The good, the bad, and the dysregulated. Exp. Neurol..

[10] Ladak A.A., Enam S.A., Ibrahim M.T. (2019). A review of the molecular mechanisms of traumatic brain injury. World Neurosurg..

[11] Giza C.C., Hovda D.A. (2001). The neurometabolic cascade of concussion. J. Athl. Train..

[12] Prins M., Greco T., Alexander D., Giza C.C. (2013). The pathophysiology of traumatic brain injury at a glance. Dis. Model. Mech..

[13] Blennow K., Hardy J., Zetterberg H. (2012). The neuropathology and neurobiology of traumatic brain injury. Neuron.

[14] Barkhoudarian G., Hovda D.A., Giza C.C. (2016). The molecular pathophysiology of concussive brain injury—An update. Phys. Med. Rehabil. Clin..

[15] McKee A.C., Robinson M.E. (2014). Military-related traumatic brain injury and neurodegeneration. Alzheimer’s Dement..

[16] Simon D.W., McGeachy M.J., Bayır H., Clark R.S., Loane D.J., Kochanek P.M. (2017). The far-reaching scope of neuroinflammation after traumatic brain injury. Nat. Rev. Neurol..

[17] Shane B. (2001). Folate chemistry and metabolism. Clin. Res. Regul. Aff..

[18] Selhub J., Morris M.S., Jacques P.F., Rosenberg I.H. (2009). Folate–vitamin B-12 interaction in relation to cognitive impairment, anemia, and biochemical indicators of vitamin B-12 deficiency. Am. J. Clin. Nutr..

[19] Ducker G., Rabinowitz J. (2017). One-carbon metabolism in health and disease. Cell Metabol..

[20] Joshi R., Adhikari S., Patro B., Chattopadhyay S., Mukherjee T. (2001). Free radical scavenging behavior of folic acid: Evidence for possible antioxidant activity. Free Radic. Biol. Med..

[21] Erel O. (2005). A new automated colorimetric method for measuring total oxidant status. Clin. Biochem..

[22] Ho P.I., Ashline D., Dhitavat S., Ortiz D., Collins S.C., Shea T.B., Rogers E. (2003). Folate deprivation induces neurodegeneration: Roles of oxidative stress and increased homocysteine. Neurobiol. Dis..

[23] Kruman I.I., Culmsee C., Chan S.L., Kruman Y., Guo Z., Penix L., Mattson M.P. (2000). Homocysteine elicits a DNA damage response in neurons that promotes apoptosis and hypersensitivity to excitotoxicity. J. Neurosci..

[24] Tisato V., Silva J.A., Longo G., Gallo I., Singh A.V., Milani D., Gemmati D. (2021). Genetics and epigenetics of one-carbon metabolism pathway in autism spectrum disorder: A sex-specific brain epigenome?. Genes.

[25] Shaikh A., Roy H. (2023). Folate deprivation induced neuroinflammation impairs cognition. Neurosci. Lett..

[26] Reynolds E. (2006). Vitamin B12, folic acid, and the nervous system. Lancet Neurol..

[27] Amirahmadi S., Hosseini M., Ahmadabady S., Akbarian M., Abrari K., Vafaee F., Rajabian A. (2021). Folic acid attenuated learning and memory impairment via inhibition of oxidative damage and acetylcholinesterase activity in hypothyroid rats. Metab. Brain Dis..

[28] Hegde V.S., Nagalli S. (2023). Leucovorin. StatPearls.

[29] Lionaki E., Ploumi C., Tavernarakis N. (2022). One-carbon metabolism: Pulling the strings behind aging and neurodegeneratin. Cells.

[30] Tommy T., Islam A.A., Hatta M., Bukhari A., Adhimarta W., Zainuddin A.A. (2021). Effect of folinic acid on serum homocysteine, TNFα, IL-10, and HMGB1 gene expression in head injury model. Ann. Med. Surg..

[31] Gristan Y.D., Patel P., Moosavi L. (2024). Folinic Acid. StatPearls.

[32] Papakostas G.I., Shelton R.C., Zajecka J.M., Etemad B., Rickels K., Clain A., Baer L., Dalton E.D., Sacco G.R., Schoenfeld D. (2012). L-methylfolate as adjunctive therapy for SSRI-resistant major depression: Results of two randomized, double-blind, parallel-sequential trials. Am. J. Psychiatry.

[33] Lam N.S.K., Long X.X., Li X., Saad M., Lim F., Doery J.C., Griffin R.C., Galletly C. (2022). The potential use of folate and its derivatives in treating psychiatric disorders: A systematic review. Biomed. Pharmacother..

[34] Macaluso M. (2022). L-methylfolate in antidepressant non-responders: The impact of body weight and inflammation. Front. Psychiatry.

[35] Gu L., Zhang L., Li C., Jiang L., Zhou J., Xie Y., Yang J., Jiang C., Zhang L., Jiang Y. (2025). Global, regional, and national burden of traumatic brain injury, 1990–2021: A systematic analysis for the global burden of disease study 2021. J. Neurotrauma.

[36] Freire M.A.M., Rocha G.S., Bittencourt L.O., Falcao D., Lima R.R., Cavalcanti J.R.L.P. (2023). Cellular and molecular pathophysiology of traumatic brain injury: What have we learned so far?. Biology.

[37] Butkova T.V., Malsagova K.A., Nakhod V.I., Petrovskiy D.V., Izotov A.A., Balakin E.I., Yurku K.A., Umnikov A.S., Pustovoyt V.I., Kaysheva A.L. (2024). Candidate molecular biomarkers of traumatic brain injury: A systematic review. Biomolecules.

[38] Jiang R., Mei S., Zhao Z. (2022). Leucovorin (folinic acid) rescue for high-dose methotrexate: A review. J. Clin. Pharm. Ther..

[39] Ibrahim A.M., Chauhan L., Bhardwaj A., Sharma A., Fayaz F., Kumar B., Alhashmi M., AlHajri N., Alam M.S., Pottoo F.H. (2022). Brain-derived neurotropic factor in neurodegenerative disorders. Biomedicines.

[40] Wang C.S., Kavalali E.T., Monteggia L.M. (2022). BDNF signaling in context: From synaptic regulation to psychiatric disorders. Cell.

[41] Bekdash R.A. (2023). Methyl donors, epigenetic alterations, and brain health: Understanding the connection. Int. J. Mol. Sci..

[42] Edman S., Horwath O., Van der Stede T., Blackwood S.J., Moberg I., Strömlind H., Nordström F., Ekblom M., Katz A., Apró W. (2024). Pro-brain-derived neurotrophic factor (BDNF), but not mature BDNF, is expressed in human skeletal muscle: Implications for exercise-induced neuroplasticity. Function.

[43] Lalonde R., Joyal C.C., Botez M.I. (1993). Effects of folic acid and folinic acid on cognitive and motor behaviors in 20-month-old rats. Pharmacol. Biochem. Behav..

[44] Rosa P.B., Ribeiro C.M., Bettio L.E., Colla A., Lieberknecht V., Moretti M., Rodrigues A.L.S. (2014). Folic acid prevents depressive-like behavior induced by chronic corticosterone treatment in mice. Pharmacol. Biochem. Behav..

[45] Taha M., Eldemerdash O.M., Elshaffei I.M., Yousef E.M., Senousy M.A. (2023). Dexmedetomidine attenuates methotrexate-induced neurotoxicity and memory deficits in rats through improving hippocampal neurogenesis: The role of miR-15a/ROCK-1/ERK1/2/CREB/BDNF pathway modulation. Int. J. Mol. Sci..

[46] Budni J., Freitas A.E., Binfaré R.W., Rodrigues A.L.S. (2012). Role of potassium channels in the antidepressant-like effect of folic acid in the forced swimming test in mice. Pharmacol. Biochem. Behav..

[47] Budni J., Zomkowski A.D., Engel D., Santos D.B., dos Santos A.A., Moretti M., Valvassori S.S., Ornell F., Quevedo J., Farina M. (2013). Folic acid prevents depressive-like behavior and hippocampal antioxidant imbalance induced by restraint stress in mice. Exp. Neurol..

[48] Zhou Y., Cong Y., Liu H. (2020). Folic acid ameliorates depression-like behaviour in a rat model of chronic unpredictable mild stress. BMC Neurosci..

[49] Sun W., Qing Q., Cheng X., Chen J., Yu N., Zhu L., Zhao M. (2022). Effects of chronic folate deficiency and sex differences on depression-like behavior in mice. Exp. Ther. Med..

[50] Brocardo P.S., Budni J., Pavesi E., Franco J.L., Uliano-Silva M., Trevisan R., Terenzi M.G., Dafre A.L., Rodrigues A.L.S. (2010). Folic acid administration prevents ouabain-induced hyperlocomotion and alterations in oxidative stress markers in the rat brain. Bipolar Disord..

[51] Singh R., Kanwar S.S., Sood P.K., Nehru B. (2011). Beneficial effects of folic acid on enhancement of memory and antioxidant status in aged rat brain. Cell. Mol. Neurobiol..

[52] Sequeira J.M., Desai A., Berrocal-Zaragoza M.I., Murphy M.M., Fernandez-Ballart J.D., Quadros E.V. (2016). Exposure to folate receptor alpha antibodies during gestation and weaning leads to severe behavioral deficits in rats: A pilot study. PLoS ONE.

[53] Desai A., Sequeira J., Quadros E. (2017). Prevention of behavioral deficits in rats exposed to folate receptor antibodies: Implication in autism. Mol. Psychiatry.

[54] Tchelingerian J.-L., Quinonero J., Booss J., Jacque C. (1993). Localization of TNFα and IL-1a immunoreactivities in striatal neurons after surgical injury to the hippocampus. Neuron.

[55] Shohami E., Novikov M., Bass R., Yamin A., Gallily R. (1994). Closed head injury triggers early production of TNFα and IL-6 by brain tissue. J. Cereb. Blood Flow Metab..

[56] Fan L., Young P.R., Barone F.C., Feuerstein G.Z., Smith D.H., McIntosh T.K. (1996). Experimental brain injury induces differential expression of tumor necrosis factor-α mRNA in the CNS. Mol. Brain Res..

[57] Shohami E., Ginis I., Hallenbeck J.M. (1999). Dual role of tumor necrosis factor alpha in brain injury. Cytokine Growth Factor Rev..

[58] Sullivan P.G., Bruce-Keller A.J., Rabchevsky A.G., Christakos S., Clair D.K.S., Mattson M.P., Scheff S.W. (1999). Exacerbation of damage and altered NF-κB activation in mice lacking tumor necrosis factor receptors after traumatic brain injury. J. Neurosci..

[59] Allan S.M., Rothwell N.J. (2001). Cytokines and acute neurodegeneration. Nat. Rev. Neurosci..

[60] Knoblach S.M., Fan L., Faden A.I. (1999). Early neuronal expression of tumor necrosis factor-α after experimental brain injury contributes to neurological impairment. J. Neuroimmunol..

[61] Dash P.K., MacH S.A., Moore A.N. (2000). Regional expression and role of cyclooxygenase-2 following experimental traumatic brain injury. J. Neurotrauma.

[62] Strauss K.I., Barbe M.F., Marshall R.M., Raghupathi R., Mehta S., Narayan R.K. (2000). Prolonged cyclooxygenase-2 induction in neurons and glia following traumatic brain injury in the rat. J. Neurotrauma.

[63] Cernak I., O’Connor C., Vink R. (2001). Activation of cyclo-oxygenase-2 contributes to motor and cognitive dysfunction following diffuse traumatic brain injury in rats. Clin. Exp. Pharmacol. Physiol..

[64] Hiskens M.I., Schneiders A.G., Fenning A.S. (2024). Selective COX-2 inhibitors as neuroprotective agents in traumatic brain injury. Biomedicines.

[65] Longhi L., Perego C., Ortolano F., Aresi S., Fumagalli S., Zanier E.R., Stocchetti N., De Simoni M.-G. (2013). Tumor necrosis factor in traumatic brain injury: Effects of genetic deletion of p55 or p75 receptor. J. Cereb. Blood Flow Metab..

[66] Perez-Polo J.R., Rea H.C., Johnson K.M., Parsley M.A., Unabia G.C., Xu G., Infante S.K., DeWitt D.S., Hulsebosch C.E. (2013). Inflammatory consequences in a rodent model of mild traumatic brain injury. J. Neurotrauma.

[67] Hall E.D., Wang J.A., Bosken J.M., Singh I.N. (2016). Lipid peroxidation in brain or spinal cord mitochondria after injury. J. Bioenerg. Biomembr..

[68] Robertson C.L., Saraswati M., Fiskum G. (2007). Mitochondrial dysfunction early after traumatic brain injury in immature rats. J. Neurochem..

[69] Song K., Li Y., Zhang H., An N., Wei Y., Wang L., Tian C., Yuan M., Sun Y., Xing Y. (2020). Oxidative stress-mediated blood-brain barrier (BBB) disruption in neurological diseases. Oxidative Med. Cell. Longev..

[70] Fesharaki-Zadeh A. (2022). Oxidative stress in traumatic brain injury. Int. J. Mol. Sci..

[71] Di Pietro V., Yakoub K.M., Caruso G., Lazzarino G., Signoretti S., Barbey A.K., Tavazzi B., Lazzarino G., Belli A., Amorini A.M. (2020). Antioxidant therapies in traumatic brain injury. Antioxidants.

[72] Conti A.C., Raghupathi R., Trojanowski J.Q., McIntosh T.K. (1998). Experimental brain injury induces regionally distinct apoptosis during the acute and delayed post-traumatic period. J. Neurosci..

[73] Lorente L., Martín M.M., Argueso M., Ramos L., Solé-Violán J., Riaño-Ruiz M., Jiménez A., Borreguero-León J.M. (2015). Serum caspase-3 levels and mortality are associated in patients with severe traumatic brain injury. BMC Neurol..

[74] Griesbach G.S., Hovda D.A., Gomez-Pinilla F. (2009). Exercise-induced improvement in cognitive performance after traumatic brain injury in rats is dependent on BDNF activation. Brain Res..

[75] Failla M.D., Juengst S.B., Arenth P.M., Wagner A.K. (2016). Preliminary associations between brain-derived neurotrophic factor, memory impairment, functional cognition, and depressive symptoms following severe TBI. Neurorehabilit. Neural Repair.

[76] Shin S.S., Dixon C.E. (2015). Alterations in cholinergic pathways and therapeutic strategies targeting cholinergic system after traumatic brain injury. J. Neurotrauma.

[77] Donat C.K., Scott G., Gentleman S.M., Sastre M. (2017). Microglial activation in traumatic brain injury. Front. Aging Neurosci..

[78] Demir D., Bektaşoğlu P.K., Koyuncuoğlu T., Kandemir C., Akakın D., Yüksel M., Çelikoğlu E., Yeğen B.Ç., Gürer B. (2019). Neuroprotective effects of mildronate in a rat model of traumatic brain injury. Injury.

[79] Bektaşoğlu P.K., Koyuncuoğlu T., Özaydın D., Kandemir C., Akakın D., Yüksel M., Gürer B., Çelikoğlu E., Yeğen B.Ç. (2023). Antioxidant and neuroprotective effects of dexpanthenol in rats induced with traumatic brain injury. Injury.

[80] Hiskens M.I., Schneiders A.G., Vella R.K., Fenning A.S. (2021). Repetitive mild traumatic brain injury affects inflammation and excitotoxic mRNA expression at acute and chronic time-points. PLoS ONE.

[81] Xue Q., Wang L., Zhao Y., Tong W., Wang J., Li G., Cheng W., Gao L., Dong Y. (2022). Cortical and subcortical alterations and clinical correlates after traumatic brain injury. J. Clin. Med..

[82] Fancy S.P., Baranzini S.E., Zhao C., Yuk D.-I., Irvine K.-A., Kaing S., Sanai N., Franklin R.J., Rowitch D.H. (2009). Dysregulation of the Wnt pathway inhibits timely myelination and remyelination in the mammalian CNS. Genes Dev..

[83] Franklin R.J., Ffrench-Constant C. (2008). Remyelination in the CNS: From biology to therapy. Nat. Rev. Neurosci..

[84] Directive E. (2010). 63/EU of the European Parliament and of the Council of 22 September 2010 on the protection of animals used for scientific purposes. Off. J. Eur. Union.

[85] Percie du Sert N., Hurst V., Ahluwalia A., Alam S., Avey M.T., Baker M., Browne W.J., Clark A., Cuthill I.C., Dirnagl U. (2020). The ARRIVE guidelines 2.0: Updated guidelines for reporting animal research. J. Cereb. Blood Flow Metab..

[86] Saghaei M. (2004). Random allocation software for parallel group randomized trials. BMC Med. Res. Methodol..

[87] Arslan E., Ordu M. (2026). Sulbutiamine Ameliorates Cognitive Impairment, Neuroinflammation, and Oxidative Stress in Rats with Cerebral Ischemia. Bratisl. Med. J..

[88] Bektaşoğlu P.K., Arıkök A.T., Ergüder B.İ., Sargon M.F., Altun S.A., Ünlüler C., Börekci A., Kertmen H., Çelikoğlu E., Gürer B. (2024). Cinnamaldehyde has ameliorative effects on rabbit spinal cord ischemia and reperfusion injury. World Neurosurg. X.

[89] Ozcan M.S., Savran M., Doguc D.K., Dogan H.K., Altintas M., Cosan S. (2024). Dexpanthenol ameliorates lipopolysaccharide-induced cardiovascular toxicity by regulating the IL-6/HIF1α/VEGF pathway. Heliyon.

[90] Xiong Y., Mahmood A., Chopp M. (2013). Animal models of traumatic brain injury. Nat. Rev. Neurosci..

[91] Kumar A., Loane D.J. (2012). Neuroinflammation after traumatic brain injury: Opportunities for therapeutic intervention. Brain Behav. Immun..

[92] Woodcock T., Morganti-Kossmann M.C. (2013). The role of markers of inflammation in traumatic brain injury. Front. Neurol..

[93] Marmarou A., Foda M.A.A.-E., Van Den Brink W., Campbell J., Kita H., Demetriadou K. (1994). A new model of diffuse brain injury in rats: Part I: Pathophysiology and biomechanics. J. Neurosurg..

[94] Ucar T., Tanriover G., Gurer I., Onal M.Z., Kazan S. (2006). Modified experimental mild traumatic brain injury model. J. Trauma Acute Care Surg..

[95] Rowe R.K., Harrison J.L., Thomas T.C., Pauly J.R., Adelson P.D., Lifshitz J. (2013). Using anesthetics and analgesics in experimental traumatic brain injury. Lab Anim..

[96] Hakan T., Toklu H.Z., Biber N., Ozevren H., Solakoglu S., Demirturk P., Aker F.V. (2010). Effect of COX-2 inhibitor meloxicam against traumatic brain injury-induced biochemical, histopathological changes and blood–brain barrier permeability. Neurol. Res..

[97] Falcon E., Maier K., Robinson S.A., Hill-Smith T.E., Lucki I. (2015). Effects of buprenorphine on behavioral tests for antidepressant and anxiolytic drugs in mice. Psychopharmacology.

[98] Ryu J., Stone P., Lee S., Payne B., Gorse K., Lafrenaye A. (2021). Buprenorphine alters microglia and astrocytes acutely following diffuse traumatic brain injury. Sci. Rep..

[99] Posadas I., Santos P., Blanco A., Muñoz-Fernández M., Ceña V. (2010). Acetaminophen induces apoptosis in rat cortical neurons. PLoS ONE.

[100] Lalert L., Ji-Au W., Srikam S., Chotipinit T., Sanguanrungsirikul S., Srikiatkhachorn A., Maneesri-le Grand S. (2020). Alterations in synaptic plasticity and oxidative stress following long-term paracetamol treatment in rat brain. Neurotox. Res..

[101] Zhao W.-X., Zhang J.-H., Cao J.-B., Wang W., Wang D.-X., Zhang X.-Y., Yu J., Zhang Y.-Y., Zhang Y.-Z., Mi W.-D. (2017). Acetaminophen attenuates lipopolysaccharide-induced cognitive impairment through antioxidant activity. J. Neuroinflammation.

[102] Labib A.Y., Ammar R.M., El-Naga R.N., El-Bahy A.A.Z., Tadros M.G., Michel H.E. (2021). Mechanistic insights into the protective effect of paracetamol against rotenone-induced Parkinson’s disease in rats: Possible role of endocannabinoid system modulation. Int. Immunopharmacol..

[103] Lalert L., Tantarungsee N., Chotipinit T., Ji-au W., Srikiatkhachorn A., Maneesri-le Grand S. (2023). Long-term paracetamol treatment impairs cognitive function and brain-derived neurotrophic factor in adult rat brain. Sci. Pharm..

[104] Öz M., Erdal H. (2024). A TNF-α inhibitor abolishes sepsis-induced cognitive impairment in mice by modulating acetylcholine and nitric oxide homeostasis, BDNF release, and neuroinflammation. Behav. Brain Res..

[105] Aslanlar D.A., Vişneci E.F., Oz M., Atalik K.E.N. (2024). N-acetylcysteine ameliorates chemotherapy-induced impaired anxiety and depression-like behaviors by regulating inflammation, oxidative and cholinergic status, and BDNF release. Behav. Brain Res..

[106] Harma M., Erel O. (2003). Increased oxidative stress in patients with hydatidiform mole. Swiss Med. Wkly..

[107] Kosecik M., Erel O., Sevinc E., Selek S. (2005). Increased oxidative stress in children exposed to passive smoking. Int. J. Cardiol..

[108] Yumru M., Savas H.A., Kalenderoglu A., Bulut M., Celik H., Erel O. (2009). Oxidative imbalance in bipolar disorder subtypes: A comparative study. Prog. Neuro-Psychopharmacol. Biol. Psychiatry.

