# Supplementary material for: Repurposing Leucovorin for Mild Traumatic Brain Injury: Evidence from Biochemical and Behavioral Outcomes in Rats
Source: Pharmaceuticals (Basel). 2026 May 30;19(6):865. doi: 10.3390/ph19060865 (PMC13304662; doi:10.3390/ph19060865)
Supplement: Supplementary file 1 [file pharmaceuticals-19-00865-s001.zip › pharmaceuticals-4288440-supplementary.pdf]

The ARRIVE reporting checklist

For checking that articles describing *in vivo* animal experiments can be understood and used by everyone

Note

If you have not used a reporting guideline before, read about how and why to use them and check whether ARRIVE is the most applicable reporting guideline for your work.

Reporting guidelines are most useful when used early in research. When writing a manuscript or application, consider using the Full Guidance where you'll see explanations and examples for each item.

After writing, demonstrate adherence by completing this checklist:

1. Specify where each item is described (see Note 1).
2. Cite this checklist (See Note 2).
3. Include your completed checklist as a supplement when submitting to a journal so that future readers can use it to find information.

	Item Description	Location (or reason for not reporting)
**Essential 10**		
1. Study Design		Graphical abstract, Materials and Methods
1a. The groups being compared	For each experiment, describe the groups being compared, including control groups. If you did not use a control group, explain why.	Sham-operated Control group (CON)<br>Mild traumatic brain injury group (mTBI)<br>Mild traumatic brain injury + leucovorin-treated group (LT)<br>**Location:** Materials and Methods Experimental Design; Fig. 1
1b. The experimental unit	Describe the experimental unit (e.g., a single animal, litter, or cage of animals).	The experimental unit was a single animal.<br>**Location:** Materials and Methods Experimental Animals / Experimental Design
2. Sample Size		
2a. Number of Experimental Units	Specify the exact number of experimental units allocated to each group, and the total number in each experiment. Also indicate the total number of animals used.	Each group consisted of *n = 7 rats* (total n = 21 animals).<br>**Location:** Materials and Methods Experimental Animals / Sample Size

2b. Sample Size Justification	Explain how the sample size was decided. Provide details of any a priori sample size calculation, if done.	Group size was determined using the **resource equation method**, which is appropriate when prior effect size estimates are unavailable and supports reduction of animal use while maintaining statistical validity.<br>Materials and methods<br>**Location:** Materials and Methods Experimental Animals / Sample Size Justification
3. Inclusion and Exclusion Criteria		
3a. Inclusion and exclusion criteria	Describe any criteria used for including or excluding animals (or experimental units) during the experiment, and data points during the analysis. Specify if these criteria were established a priori. If no criteria were set, state this explicitly.	All healthy adult male Wistar albino rats meeting predefined age and weight criteria were included. No exclusion criteria were predefined.**Location:** Materials and Methods Experimental Animals
3b. Exclusions and Attritions	For each experimental group, report any animals, experimental units, or data points not included in the analysis and explain why. If there were no exclusions, state so.	No animals or data points were excluded from the analysis.**Location:** Results General Toxicity and Body Weight Changes
3c. Numbers analysed	For each analysis, report the exact value of *n* in each experimental group.	All animals allocated to each group were included in all analyses.<br>**Location:** Results General Toxicity and Body Weight Changes
4. Randomisation		
4a. Randomisation Use	State whether randomisation was used to allocate experimental units to control and treatment groups. If done, provide the method used to generate the randomisation sequence.	Animals were randomly assigned to groups using Random Allocation Software (RAS).<br>**Location:** Materials and Methods Experimental Animals / Randomisation
4b. Confounders	Describe the strategy used to minimise potential confounders such as the order of treatments and measurements, or animal/cage location. If confounders were not controlled, state this explicitly.	Potential confounders were minimized by standardizing housing conditions, surgical procedures, behavioral testing order, and timing of biochemical analyses.

		**Location:** Materials and Methods / Experimental Animals / Housing and Procedures
5. Blinding/Masking	Describe who was aware of the group allocation at the different stages of the experiment (during the allocation, the conduct of the experiment, the outcome assessment, and the data analysis).	Investigators conducting behavioral tests, biochemical assays, histopathological scoring, immunohistochemical analyses, and statistical analyses were blinded to group allocation.<br>**Location:** Materials and Methods / Immunohistochemistry; Statistical Analysis
6. Outcome Measures		
6a. Outcome Measures	Clearly define all outcome measures assessed (e.g., cell death, molecular markers, or behavioural changes).	Outcome measures included behavioral tests (OF, EPM, NOR, FST), oxidative stress indices (TAS, TOS, OSI), inflammatory markers (TNF-α, COX-2), apoptotic signaling (caspase-3), neuroplasticity and cholinergic markers (BDNF, AChE), and histopathological and immunohistochemical findings.<br>**Location:** Materials and Methods/Behavioral Assessments; Biochemical Analyses; Histopathology; Immunohistochemistry
6b. Primary Outcome Measure	For hypothesis-testing studies, specify the primary outcome measure, i.e., the outcome measure that was used to determine the sample size.	Primary outcomes were cognitive performance (NOR), biochemical markers of secondary injury, and histopathological injury scores.<br>**Location:** Materials and Methods /Outcome Measures; Results (Sections 3.2–3.4)
7. Statistical Methods		
7a. Statistical Methods used for each Analysis	Provide details of the statistical methods used for each analysis, including software used.	Statistical analyses were performed using IBM SPSS Statistics v27.0. One-way

		ANOVA or Kruskal–Wallis tests were applied as appropriate, with suitable post hoc tests. **Location:** Materials and Methods / Statistical Analysis
7b. Statistical Assumptions	Describe any methods used to assess whether the data met the assumptions of the statistical approach, and what was done if the assumptions were not met.	Normality was assessed using Q–Q plots and Shapiro–Wilk tests. Non-parametric tests were applied when assumptions were violated. **Location:** Materials and Methods /Statistical Analysis
8. Experimental Animals		
8a. Species-appropriate Details	Provide species-appropriate details of the animals used, including species, strain and substrain, sex, age or developmental stage, and, if relevant, weight.	Adult (10 weeks old) male Wistar albino rats (200–300 g) were used. **Location:** Materials and Methods / Experimental Animals
8b. Further Information	Provide further relevant information on the provenance of animals, health/immune status, genetic modification status, genotype, and any previous procedures.	Animals were obtained from the accredited KONÜDAM breeding facility, were pathogen-free, and had no prior experimental exposure. **Location:** Materials and Methods /Experimental Animals
9. Experimental Procedures		
9a. What was done	What was done, how it was done, and what was used.	mTBI was induced using a modified Marmarou weight-drop model; leucovorin (20 mg/kg, i.p.) was administered post-injury. **Location:** Materials and Methods / Mild Brain Trauma Model; Experimental Design
9b. When and how often procedures were conducted	For each experimental group, including controls, describe **when and how often** procedures were performed.	Trauma induction and treatment were performed once; behavioral and biochemical assessments followed predefined post-injury timelines. **Location:** Materials and Methods / Experimental Design; Behavioral Assessments

9c. Where procedures were conducted	For each experimental group, including controls, describe **where** procedures were conducted (including detail of any acclimatisation periods).	All procedures were conducted at the KONÜDAM Experimental Medicine Application and Research Center after acclimatization. **Location:** Materials and Methods / Experimental Animals / Housing
9d. Why procedures were done	For each experimental group, including controls, describe **why** procedures were conducted.	Procedures were conducted to evaluate the neuroprotective effects of leucovorin against mTBI-induced secondary injury. **Location:** Introduction; Materials and Methods /Experimental Design
10. Results		
10a. Summary/Descriptive Statistics per group	For each experiment conducted, including independent replications, report a summary/descriptive statistics for each experimental group, with a measure of variability where applicable (e.g., mean and SD, or median and range).	Results are presented as mean ± SD or median (min–max), as appropriate. **Location:** Results; Figure Legends
10b. Effect sizes and confidence intervals	For each experiment conducted, including independent replications, report the effect size with a confidence interval, if applicable.	Effect sizes were calculated where appropriate; confidence intervals were not routinely reported. **Location:** Statistical Analysis; Results
**Recommended Set**		
11. Abstract	Provide an accurate summary of the research objectives, animal species, strain and sex, key methods, principal findings, and study conclusions.	The abstract accurately summarizes objectives, animal model, methods, key findings, and conclusions. **Location:** Abstract
12. Background		
12a. Rationale	Include sufficient scientific background to understand the rationale and context for the study, and explain the experimental approach.	The study rationale is based on secondary injury mechanisms in mTBI and folate-dependent metabolic modulation. **Location:** Introduction
12b. Species and model	Explain how the animal species and model used address the scientific objectives and, where appropriate, the relevance to human biology.	The rat mTBI weight-drop model was selected for translational relevance to human concussion.

		**Location:** Introduction; Materials and Methods/Mild Brain Trauma Model
13. Objectives	Clearly describe the research question, research objectives and, where appropriate, specific hypotheses being tested.	To evaluate whether leucovorin modulates secondary injury mechanisms and improves behavioral, biochemical, and histopathological outcomes after mTBI.<br>**Location:** Introduction (final paragraph)
14. Ethical statement	Provide the name of the ethical review committee or equivalent that has approved the use of animals in this study and any relevant licence or protocol numbers (if applicable). If ethical approval was not sought or granted, provide a justification.	All procedures were approved by the KONÜDAM Institutional Animal Ethics Committee (Approval No: 2024-21).<br>**Location:** Materials and Methods/ Ethics Statement
15. Housing and husbandry	Provide details of housing and husbandry conditions, including any environmental enrichment.	Animals were housed under controlled temperature and humidity with a 12-h light/dark cycle and free access to food and water.<br>**Location:** Materials and Methods → Experimental Animals
16. Animal Care and Monitoring		
16a. Reducing pain, suffering, and distress	Describe any interventions or steps taken in the experimental protocols to reduce pain, suffering, and distress.	All surgical procedures were performed under ketamine/xylazine anesthesia.<br>**Location:** Materials and Methods / Mild Brain Trauma Model
16b. Adverse events	Report any expected or unexpected adverse events.	No unexpected adverse events were observed.<br>**Location:** Results → General Toxicity and Body Weight Changes
16c. Humane endpoints	Describe the humane endpoints established for the study, the signs that were monitored, and the frequency of monitoring. If the study did not set humane endpoints, state this.	Animals were monitored daily; no humane endpoints were reached.**Location:** Materials and Methods /Ethics / Animal Monitoring
17. Interpretation/ scientific implications		

17a. Interpretation/scientific implications	Interpret the results, taking into account the study objectives and hypotheses, current theory, and other relevant studies in the literature.	Results indicate leucovorin constrains secondary injury cascades after mTBI.<br>**Location:** Discussion
17b. Limitations	Comment on the study limitations, including potential sources of bias, limitations of the animal model, and imprecision associated with the results.	Limitations include single-dose administration and a single injury model.<br>**Location:** Discussion /Limitations paragraph
18. Generalisability/translation	Comment on whether, and how, the findings of this study are likely to generalise to other species or experimental conditions, including any relevance to human biology (where appropriate).	Findings support further translational investigation of leucovorin in traumatic brain injury.<br>**Location:** Discussion; Conclusion
19. Protocol registration	Provide a statement indicating whether a protocol (including the research question, key design features, and analysis plan) was prepared before the study, and if and where this protocol was registered.	No protocol was registered prior to the study.<br>**Location:** Methods /Protocol Registration (statement)
20. Data Access	Provide a statement describing if and where study data are available.	Data are available from the corresponding author upon reasonable request.<br>**Location:** Supplementary Data / Data Availability Statement
21. Declaration of interests		
21a. Conflicts of interests	Declare any potential conflicts of interest, including financial and nonfinancial. If none exist, this should be stated.	The authors declare no conflicts of interest.<br>**Location:** Conflict of Interest Statement
21b. Funding	List all funding sources (including grant identifier) and the role of the funder(s) in the design, analysis, and reporting of the study.	No external funding was received.<br>**Location:** Funding Statement

1 How to specify where content is

Tell the reader where they can find information. E.g.,

- Results; paragraph 2
- Methods, Participants; paragraphs 1 & 2.
- Table 3
- Supplement B, para. 4

If you have chosen not to describe an item, explain why. You can do this in the checklist, or as a note below it.

You can describe items in the article body, or in tables, figures, or supplementary materials, and should prioritize items you feel are most important to your intended audience. The order of items in your manuscript does not need to match the order of items in this checklist. You can decide how best to structure your work.

2 How to cite

Describe how you used ARRIVE at the end of your Methods section, referencing the resources you used e.g.,

> ‘We used the ARRIVE reporting guideline(1) to draft this manuscript, and the ARRIVE reporting checklist(2) when editing, included in supplement A’

If you use a reporting checklist, remember to include it as a supplement when publishing so that readers can easily find information and see how you have interpreted the guidance.

1. Sert NP, Hurst V, Ahluwalia A, Alam S, Avey MT, Baker M, et al. The ARRIVE guidelines 2.0: Updated guidelines for reporting animal research. PLOS Biology [Internet]. 2020 Jul;18(7):e3000410. Available from: https://journals.plos.org/plosbiology/article?id=10.1371/journal.pbio.3000410

2. Sert NP, Hurst V, Ahluwalia A, Alam S, Avey MT, Baker M, et al. The ARRIVE reporting checklist. In: Harwood J, Albury C, Beyer J de, Schlüssel M, Collins G, editors. The EQUATOR network reporting guideline platform [Internet]. The UK EQUATOR Centre; 2025. Available from: https:/resources.equator-network.org/reporting-guidelines/arrive/arrive-checklist.docx
